# Mechanistic drivers of PD-L1/CTLA-4 checkpoint inhibitor–associated immune toxicity and systemic organ injury

**DOI:** 10.3389/fonc.2026.1808024

**Published:** 2026-04-23

**Authors:** Junfeng Chen, Lin Zhang, Minqqiang Gao, Siqing Min

**Affiliations:** Center of Emergency & Intensive Care Unit, Jinshan Hospital, Fudan University, Shanghai, China

**Keywords:** immune checkpoint inhibitors, immune-related toxicity, mitochondrial dysfunction, multi-organ injury, pyroptosis

## Abstract

**Background:**

Immune checkpoint inhibitors targeting PD-1/PD-L1 and CTLA-4 have substantially improved outcomes in multiple malignancies, but they may also trigger dysregulated immune activation accompanied by systemic toxic effects and multi-organ injury. Although immune-related adverse events are increasingly recognized in oncology practice, the clinical architectures through which immune toxicity progresses across organ systems, and the biological pathways that may support earlier risk assessment and mechanism-informed intervention, remain insufficiently defined.

**Methods:**

We performed a translational multicohort study integrating clinical phenotyping, biomarker modeling, and mechanistic validation. In a development cohort derived from a large United States electronic health record database, cross-organ co-occurrence networks were used to characterize reproducible patterns of organ involvement, and data-driven phenotypes were derived using non-negative matrix factorization and hierarchical clustering, followed by landmark outcome analyses. A biomarker-informed risk framework integrating immune-toxicity features with conventional severity indices was subsequently evaluated in an independent United States external validation cohort. To assess biological concordance across clinical and experimental levels, parallel analyses were conducted in biospecimens from a prospectively enrolled single-center Chinese patient cohort and in preclinical models, with emphasis on pyroptosis-related signaling, myeloid inflammatory activation, endothelial–coagulation coupling, and mitochondrial injury.

**Results:**

Immune-toxicity-associated systemic injury demonstrated stable cross-system architectures that segregated into clinically interpretable phenotypes with graded mortality risk, organ-support requirements, and recovery trajectories. Compared with conventional severity assessment alone, incorporation of immune-toxicity biomarker axes improved risk discrimination and supported more refined patient stratification across cohorts. Mechanistic analyses further identified prominent activation of pyroptosis-related pathways together with mitochondrial stress, while myeloid-dominated inflammation and endothelial activation were associated with propagation of systemic injury across organs. These findings linked phenotypic heterogeneity at the clinical level with biologically plausible injury programs observed in patient biospecimens and experimental systems.

**Conclusions:**

This study establishes a translational framework connecting clinical phenotypes, biomarker-based risk stratification, and biologically supported injury pathways in immune checkpoint inhibitor-associated systemic toxicity. The findings may support earlier recognition and structured clinical assessment of patients with suspected immune-toxicity-associated organ injury and provide a rationale for future mechanism-guided intervention studies. Further multicenter, diagnostically controlled, and ethnically diverse investigations are needed to determine the generalizability of these findings and their utility in distinguishing immune-toxicity-associated critical illness from other causes of systemic inflammation and organ failure.

## 1 Introduction

Immune checkpoint inhibitors targeting PD-1/PD-L1 and CTLA-4 have reshaped the treatment landscape of advanced malignancies over the past decade. By interrupting signaling pathways that enable tumor immune escape, these agents have produced durable clinical benefit across multiple cancer types (1, 2). At the same time, the immune activation required for antitumor efficacy may also disrupt immune homeostasis and provoke immune-related toxic effects involving several organ systems. This risk becomes more pronounced under dual-checkpoint strategies, in which augmented antitumor activity is accompanied by a higher frequency of severe toxicity. As the indications for immunotherapy continue to expand, clinicians are increasingly confronted with patients who deteriorate rapidly after checkpoint blockade and require intensive monitoring or critical care support (3).

Most existing clinical evidence and management guidelines for immune-related adverse events have been developed from general oncology settings rather than from critically ill populations (4). As a result, the dominant clinical framework remains largely centered on single-organ toxicities, such as myocarditis, pneumonitis, hepatitis, or colitis. In intensive care settings, however, patients often present with concurrent dysfunction across multiple organs, and their trajectories are shaped not only by the severity of injury within each organ but also by interactions among organ systems. In this context, multi-organ dysfunction syndrome reflects a coordinated pathophysiological process rather than a simple accumulation of isolated adverse events. Conventional single-organ assessment models are therefore insufficient to capture the evolving, system-level architecture of immune-toxicity-associated critical illness (5, 6). A broader framework that accounts for cross-organ coupling may provide a more clinically meaningful basis for risk assessment and management in these patients.

A further challenge arises from the overlap between immune-toxicity-associated systemic injury and other inflammatory states commonly encountered in critical care, particularly infection-related or trauma-related syndromes (7). These conditions may share fever, hemodynamic instability, respiratory deterioration, leukocyte abnormalities, and fluctuating inflammatory mediators, making bedside interpretation difficult. Although this overlap creates substantial diagnostic uncertainty, the therapeutic implications are fundamentally different. Infection-directed critical illness requires prompt antimicrobial control and source management, whereas severe immune checkpoint inhibitor-associated toxicity often necessitates rapid immunosuppression, including corticosteroids or other targeted immunomodulatory strategies (8). In practice, delayed recognition of immune-mediated toxicity may allow irreversible organ deterioration, while premature immunosuppression in the setting of unrecognized infection may also be harmful. For this reason, there is a pressing need for clinically grounded frameworks that can support earlier recognition, structured risk stratification, and biologically informed adjudication of suspected immune-toxicity-associated organ injury in critically ill oncology patients (9).

Another unresolved issue is that the biological processes linking checkpoint inhibitor exposure to coordinated multi-organ dysfunction remain incompletely defined. Prior work has identified important components of immune-related toxicity, including cytokine activation, myeloid cell involvement, endothelial perturbation, and tissue-specific inflammatory injury. However, these observations have rarely been integrated into a unified model that connects clinical heterogeneity, cross-organ injury patterns, and experimentally testable mechanisms. This gap limits progress in two ways. First, it constrains the development of clinically useful phenotyping strategies for patients whose deterioration does not follow a single-organ pattern. Second, it hampers efforts to identify mechanism-informed intervention windows that extend beyond nonspecific supportive care.

To address these limitations, the present study used a translational multicohort design that combined clinical phenotyping, biomarker-based modeling, and mechanistic validation. We sought to characterize reproducible patterns of organ involvement after PD-1/PD-L1 and/or CTLA-4 blockade, define clinically interpretable phenotypes associated with differential outcomes, and evaluate whether immune-toxicity-related biomarker axes improve risk stratification beyond conventional severity assessment (10). We further examined whether these clinical patterns correspond to biologically plausible injury programs in patient biospecimens and experimental models, with particular attention to pyroptosis-related signaling, myeloid-dominated inflammation, endothelial–coagulation coupling, and mitochondrial stress. Rather than claiming definitive diagnostic separation from all other causes of critical illness, this study aims to provide a structured framework for understanding phenotypic heterogeneity and mechanistic convergence in immune checkpoint inhibitor-associated systemic toxicity.

## 2 Methods

### 2.1 Study design

This study used a translational multicohort design integrating retrospective clinical phenotyping, biomarker-based risk modeling, and mechanistic validation. The clinical component consisted of two independent United States electronic health record cohorts. The development cohort was used for phenotype discovery and model derivation, and the external validation cohort was used to assess reproducibility and transportability. The mechanistic component consisted of a prospectively enrolled single-center Chinese biospecimen cohort from Jinshan Hospital, Fudan University, together with complementary *in vitro* and *in vivo* experiments. This design was intended to connect clinical heterogeneity with biologically plausible injury pathways rather than to perform direct patient-level harmonization across countries. Instead, the study adopted a phenotype-to-mechanism translational alignment strategy, in which reproducible clinical patterns identified in the United States cohorts were examined for biological concordance in the Chinese biospecimen cohort and experimental systems.

The clinical arm was designed to characterize cross-organ co-occurrence structures of immune-toxicity-associated critical illness, derive clinically interpretable phenotypes, and develop prediction models for early multiple organ dysfunction syndrome (MODS) after intensive care unit (ICU) admission. The mechanistic arm was designed to evaluate pyroptosis-related signaling, myeloid inflammatory amplification, endothelial–coagulation coupling, and mitochondrial dysfunction as candidate pathways underlying systemic organ injury. This framework was intended to support structured risk stratification and biological interpretation rather than definitive diagnostic separation of immune toxicity from all other causes of critical illness.

ICU admission served as time zero for all time-dependent analyses. A fixed-window dynamic framework was applied, with predictors extracted from the first 24 h and 48 h after ICU admission and the primary clinical endpoint defined as MODS within 72 h. These windows were chosen to capture the early phase of physiologic deterioration while preserving a clinically actionable interval for escalation of monitoring and intervention. Landmark analyses were performed at 48 h and 72 h after ICU admission to reduce time-dependent bias in phenotype-specific outcome comparisons. The development cohort included 428 critically ill patients, and the external validation cohort included 176 critically ill patients. The mechanistic biospecimen cohort comprised 30 consecutively enrolled patients.

### 2.2 Study population and case adjudication

Eligible patients in the clinical cohorts were adults aged 18 years or older with solid malignancies who were admitted to the ICU after recent exposure to anti–PD-(L)1 therapy, anti–CTLA-4 therapy, or combined checkpoint blockade. Exposure eligibility was defined as receipt of immune checkpoint inhibitor treatment within 90 days before ICU admission. This interval was selected to preserve treatment traceability while covering the period in which severe immune-related toxicity remains clinically plausible. Patients were required to have sufficient early ICU data to support dynamic feature extraction, including vital signs, key laboratory variables, and organ-support records within the first 48 h after ICU admission. The development and validation cohorts were restricted to patients with complete documentation of recent ICI exposure, cancer type, ICU admission context, and early physiologic course.

Patients were excluded if they received comfort-focused care without active organ support, had irreversible end-stage multi-organ failure already present at ICU entry such that temporal attribution could not be assessed, or lacked critical variables required for the primary analyses. Patients with hematologic malignancies, post-transplant immune complications, or insufficient medication traceability were also excluded from the primary modeling datasets in order to preserve etiologic consistency.

Because critically ill patients often present with overlapping inflammatory syndromes, suspected immune-toxicity-associated organ injury was adjudicated using a structured multistep process rather than a single chart label. Initial screening incorporated clinical presentation, laboratory abnormalities, radiographic findings, and the temporal relationship between recent ICI exposure and organ dysfunction. Secondary adjudication incorporated multidisciplinary consultation records from oncology, critical care, cardiology, pulmonology, or infectious disease teams when available, together with response to corticosteroids or other immunomodulatory treatment and re-evaluation of competing explanations such as infection, tumor progression, thromboembolic events, or other treatment-related injury. Infection-related variables, including microbiological cultures, procalcitonin, escalation of antimicrobial therapy, and formal sepsis documentation, were recorded systematically and incorporated into sensitivity and subgroup analyses. This adjudication process was intended to reduce misclassification and support structured clinical interpretation, but it was not designed to establish a definitive diagnostic test distinguishing immune toxicity from infection in all cases.

For the mechanistic validation cohort, 30 consecutive critically ill cancer patients with recent ICI exposure who were managed at Jinshan Hospital between March 2023 and October 2025 were prospectively enrolled after ethics approval and written informed consent. Enrollment required anticipated ICU stay of at least 48 h and the feasibility of serial blood collection at prespecified time points. This cohort was used for temporal plasma and peripheral blood mononuclear cell profiling and was not used for phenotype discovery or predictive model training.

### 2.3 Variables and feature engineering

Variables were organized into five prespecified domains: baseline patient characteristics, cancer and treatment exposure, organ dysfunction severity, inflammation–coagulation biology, and ICU process-of-care variables. Baseline variables included age, sex, body mass index, smoking status, major comorbidities, Eastern Cooperative Oncology Group performance status, tumor type, disease stage, and prior lines of systemic therapy. Treatment exposure variables included ICI class, monotherapy versus combination therapy, interval from last ICI administration to ICU admission, prior immune-related adverse events, and recent systemic corticosteroid or other immunosuppressive treatment. The tumor spectrum was dominated by lung cancer, with additional gastrointestinal, hepatobiliary or pancreatic, genitourinary, melanoma or skin, head and neck, and other solid tumors represented in both clinical cohorts.

Severity-of-illness variables included APACHE II, total SOFA score, and organ-specific SOFA subcomponents. ICU support variables included invasive mechanical ventilation, vasoactive drug use, renal replacement therapy, oxygenation support intensity, and early treatment escalation patterns. Laboratory variables were grouped into inflammatory, endothelial–coagulation, and organ-injury domains. Inflammatory variables included leukocyte count, neutrophil fraction, C-reactive protein, and interleukin-6 when available. Endothelial–coagulation variables included platelet count, D-dimer, fibrinogen, prothrombin time, activated partial thromboplastin time, and soluble thrombomodulin in the mechanistic cohort. Organ-injury variables included lactate, lactate clearance, cardiac troponin I, N-terminal pro–B-type natriuretic peptide, alanine aminotransferase, total bilirubin, creatinine, estimated glomerular filtration rate, and arterial blood gas measures.

To preserve interpretability while capturing temporal dynamics, candidate features were engineered using clinically transparent rules within the predefined prediction windows. For continuous variables, we derived first recorded values, peak values, nadir values, absolute change, relative change, slopes, and burden measures. For organ-support variables, we derived utilization status, initiation timing, cumulative duration, maximum dose, and escalation indicators, because these represent downstream but clinically informative expressions of evolving severity. All predictors were restricted to the corresponding 24 h or 48 h window to avoid information leakage. Missing data were handled using multiple imputation by chained equations for variables with less than 25% missingness. Variables with more extensive missingness or limited clinical interpretability were excluded from the primary models. Sensitivity analyses compared complete-case and imputed analyses.

### 2.4 Co-occurrence analysis and phenotype discovery

To characterize the systemic structure of immune-toxicity-associated organ injury, we first performed co-occurrence analyses across clinically defined immune-toxicity events and organ dysfunction domains. Immune-toxicity events included suspected pneumonitis, myocarditis, hepatitis, nephritis, colitis, encephalopathy, severe dermatologic toxicity, and endocrine crisis. Organ dysfunction domains included respiratory failure, circulatory failure, renal dysfunction, hepatic dysfunction, myocardial injury, coagulation disturbance, neurologic dysfunction, and metabolic instability. Pairwise associations were quantified using φ coefficients for binary variables and Spearman correlation coefficients for ordinal or continuous burden measures. Network representations were then constructed to identify highly connected domains and bridging nodes. False-discovery control was performed using the Benjamini–Hochberg procedure with a q value threshold of 0.10.

Phenotype discovery was performed using non-negative matrix factorization followed by hierarchical clustering. Input variables included organ dysfunction measures, inflammatory indices, endothelial–coagulation variables, and early organ-support features extracted within 48 h after ICU admission. Variables were standardized to nonnegative scaled matrices, and stability analyses were conducted over 200 repeated runs with random initialization. The number of phenotypes was determined by jointly considering cophenetic correlation, reconstruction error, silhouette performance, consensus stability, and clinical interpretability. The final solution retained four phenotypes, which were then labeled according to their dominant clinical signatures. This four-group structure was consistent with the graded patterns of MODS, mortality, organ-support burden, and ICU-free days observed in both the development and external validation cohorts.

Phenotype–outcome associations were examined using landmark analyses to reduce bias from early death, early treatment escalation, or differential observation time. Primary and secondary outcomes included MODS within 72 h, 28-day mortality, ICU-free days, ICU length of stay, and organ-support burden. Reproducibility of phenotypic gradients was then examined in the external validation cohort.

### 2.5 Predictive model development and validation

The prediction framework was designed to maximize bedside relevance, interpretability, and external transportability. ICU admission was defined as time zero. Candidate predictors were extracted exclusively from the first 24 h or 48 h after ICU admission, and the primary target was development of MODS within 72 h. Model construction followed a hierarchical strategy intended to quantify the incremental value of immune-toxicity-related information beyond standard critical care assessment.

The baseline model included established severity indicators and core clinical covariates, including total SOFA score, APACHE II score, age, metastatic disease status, tumor type, baseline organ-support intensity, recent corticosteroid exposure, and structured indicators of possible infection. Augmented models then incorporated immune-toxicity-related variables spanning inflammatory activation, endothelial–coagulation perturbation, organ-injury biomarkers, recent ICI exposure patterns, prior immune-related adverse events, and early immunosuppressive treatment signals. This layered strategy allowed formal comparison of conventional severity assessment alone versus severity assessment supplemented by immune-toxicity-related axes.

Penalized multivariable logistic regression was used as the primary interpretable model, and gradient boosting was used as a complementary nonlinear approach. The regression model used least absolute shrinkage and selection operator regularization with 10-fold cross-validation to select the tuning parameter. The gradient-boosting model used a maximum tree depth of 3, learning rate of 0.03, subsampling fraction of 0.8, and 300 boosting iterations, followed by isotonic calibration. Feature selection balanced clinical plausibility, statistical stability, and collinearity control.

Model performance was evaluated using area under the receiver operating characteristic curve, area under the precision–recall curve, Brier score, calibration slope, calibration intercept, and decision-curve analysis. Incremental value was assessed by comparing baseline and augmented models using changes in area under the curve, net reclassification improvement, and integrated discrimination improvement. Internal validation used 1000 bootstrap resamples. External validation was performed in the independent validation cohort. Performance heterogeneity across tumor type, ICI regimen, and infection-probability strata was examined to evaluate transportability. Predicted probabilities were then translated into three clinically interpretable risk strata: low risk, below 0.20; intermediate risk, 0.20 to 0.49; and high risk, 0.50 or greater. These strata were intended to support escalation of surveillance, multidisciplinary review, and timely immune-toxicity-directed assessment.

### 2.6 Mechanistic validation strategy

Mechanistic analyses were designed to test whether the clinical phenotypes and biomarker-derived risk signals were biologically concordant with experimentally measurable injury programs. The primary mechanistic domains were pyroptosis-related cell-death signaling, myeloid-dominated inflammatory amplification, endothelial–coagulation activation, and mitochondrial dysfunction. Because MODS may involve multiple regulated cell-death pathways, the study did not assume pyroptosis to be the exclusive initiating mechanism. Instead, pyroptosis was prioritized as a leading experimentally tractable pathway based on convergent signals from clinical biomarker patterns, PBMC analyses, tissue injury readouts, and intervention experiments. This strategy was intended to identify dominant and actionable mechanisms rather than to exclude necroptosis, ferroptosis, or other forms of cell death.

For the human mechanistic cohort, peripheral blood was collected at 0–2 h, 24 h, 48 h, and 72 h after ICU admission. At each time point, 10 mL of whole blood was collected into EDTA tubes for plasma and PBMC isolation and 5 mL into serum-separation tubes for biochemical assays. Samples were processed within 2 h of collection. Plasma was isolated by centrifugation at 1500 × g for 15 min at 4 °C and stored at −80 °C. PBMCs were isolated by density-gradient centrifugation using Ficoll and either analyzed immediately or cryopreserved in 90% fetal bovine serum and 10% dimethyl sulfoxide.

Plasma assays quantified inflammatory cytokines, cell-death-related mediators, endothelial activation markers, coagulation indices, and neutrophil extracellular trap-related signals. The analyte panel included interleukin-1β, interleukin-6, interleukin-18, tumor necrosis factor-α, HMGB1, soluble TREM1, soluble thrombomodulin, angiopoietin-2, von Willebrand factor, tissue factor, D-dimer, cell-free DNA, and myeloperoxidase–DNA complexes. PBMC phenotyping by flow cytometry evaluated CD3, CD4, CD8, PD-1, TIM-3, HLA-DR, CD14, CD16, CD11b, CD66b, active caspase-1, and propidium iodide uptake or 7-AAD uptake to estimate membrane permeabilization and inflammatory cell-death signatures. Associations between these markers and clinical outcomes were examined with adjustment for baseline severity and infection probability.

For immunoblotting in the human cohort, PBMC lysates were used to assess full-length and cleaved caspase-1, GSDMD, GSDME, NLRP3, ASC, cleaved IL-1β, and β-actin. A subset of plasma samples was also applied to cultured human cells for functional assays. In these experiments, human induced pluripotent stem cell-derived cardiomyocytes, human umbilical vein endothelial cells, and THP-1-derived macrophages were incubated with 10% patient plasma for 24 h. Endpoints included lactate dehydrogenase release, propidium iodide uptake, caspase-1 activity, mitochondrial membrane potential by JC-1 staining, mitochondrial reactive oxygen species by MitoSOX, ATP content, oxygen-consumption-related metabolic readouts, transendothelial electrical resistance, FITC-dextran permeability, ICAM-1 expression, and VCAM-1 expression.

**Figure 1:**
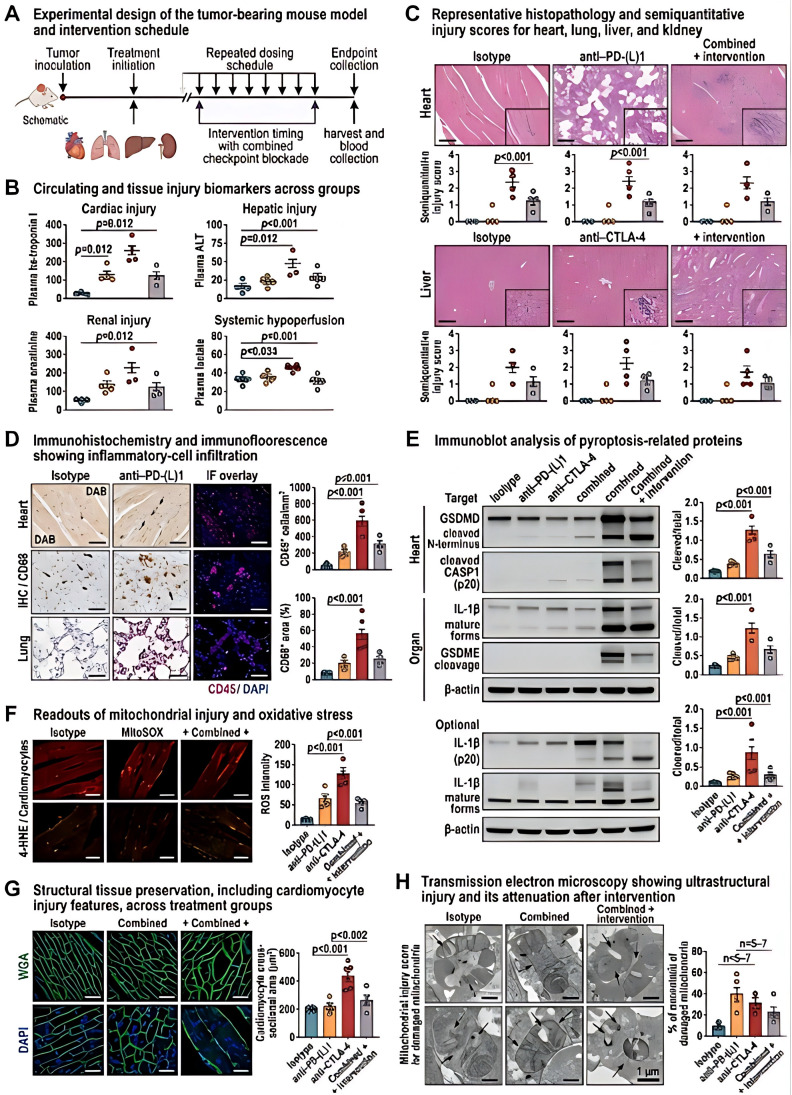
Preclinical validation showing attenuation of checkpoint-blockade-associated organ injury by targeting pyroptosis-related signaling and mitochondrial stress. **(A)** Experimental design of the tumor-bearing mouse model and intervention schedule. Tumor-bearing mice received isotype control, anti–PD-(L)1, anti–CTLA-4, combined anti–PD-(L)1 plus anti–CTLA-4 treatment, or combined treatment with pathway-directed intervention. **(B)** Circulating and tissue injury biomarkers across groups. **(C)** Representative histopathology and semiquantitative injury scores for heart, lung, liver, and kidney. **(D)** Immunohistochemistry and immunofluorescence showing inflammatory-cell infiltration. **(E)** Immunoblot analysis of pyroptosis-related proteins. The immunoblots were performed on tissue lysates and compartment-enriched fractions as specified in the Methods. **(F)** Readouts of mitochondrial injury and oxidative stress. **(G)** Structural tissue preservation, including cardiomyocyte injury features, across treatment groups. **(H)** Transmission electron microscopy showing ultrastructural injury and its attenuation after intervention. Inhibitors used in this figure targeted distinct nodes within the pyroptosis–mitochondrial injury axis: disulfiram was used to suppress gasdermin pore formation, VX-765 to inhibit caspase-1 activity, MCC950 to inhibit NLRP3 inflammasome activation, and MitoTEMPO to reduce mitochondrial reactive oxygen species. Statistical analyses were performed using one-way ANOVA with *post hoc* multiple-comparison correction or Kruskal–Wallis testing, as appropriate.

Pathway inhibition experiments were performed to assess causal contribution. The pyroptosis inhibitor disulfiram was used at 10 μM, the caspase-1 inhibitor VX-765 at 20 μM, the NLRP3 inhibitor MCC950 at 5 μM, and the mitochondrial reactive oxygen species scavenger MitoTEMPO at 10 μM. Inhibitors were added 1 h before plasma exposure. Vehicle-treated cells served as matched controls. These intervention arms were used to support the interpretation of **Figures 1B–F** and to define the target of each inhibitor within the pyroptosis–mitochondrial injury axis.

For *in vivo* validation, female C57BL/6 mice aged 6–8 weeks were used. Tumor-bearing mice were established by subcutaneous inoculation of 5 × 10^5 Lewis lung carcinoma cells into the right flank. When tumors reached approximately 80–120 mm^3, mice were randomized into the following groups: tumor-bearing control with isotype antibodies, anti–PD-L1 treatment, anti–CTLA-4 treatment, combined anti–PD-L1 plus anti–CTLA-4 treatment, combined treatment plus pyroptosis-pathway inhibition, and combined treatment plus mitochondrial-targeted intervention. In selected experiments, naïve non–tumor-bearing mice were included as physiological controls to distinguish tumor-related baseline inflammation from treatment-associated systemic injury. Checkpoint-blocking antibodies were administered intraperitoneally every 3 days for a total of four doses at 200 μg per mouse for anti–PD-L1 and 100 μg per mouse for anti–CTLA-4. Disulfiram was administered by oral gavage at 50 mg/kg daily, and MitoTEMPO was administered intraperitoneally at 5 mg/kg daily during the intervention phase.

Cardiac function was evaluated by transthoracic echocardiography, including left ventricular ejection fraction and fractional shortening. Serum and tissue injury endpoints included troponin I, creatine kinase-MB, alanine aminotransferase, creatinine, lactate, and D-dimer. Histopathology was performed in heart, lung, liver, and kidney using hematoxylin and eosin staining, Masson staining where appropriate, immunohistochemistry, and immunofluorescence. Tissue-level readouts included Ly6G-positive neutrophil infiltration, CD68-positive myeloid infiltration, VCAM-1, ICAM-1, cleaved caspase-1, GSDMD-N, GSDME-N, 4-HNE, TOM20, and TUNEL-compatible injury patterns. Ultrastructural changes were evaluated using transmission electron microscopy.

**Figure 2:**
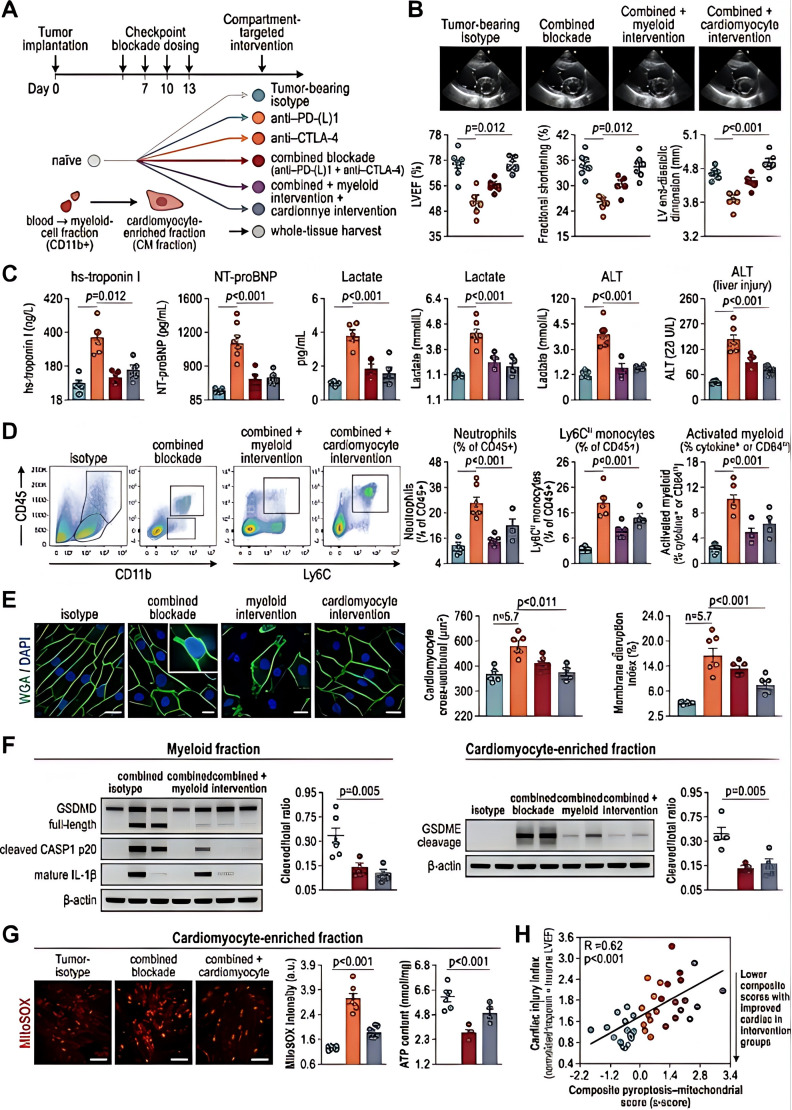
Compartment-resolved analyses identify myeloid and cardiomyocyte injury programs contributing to checkpoint-blockade-associated MODS. **(A)** Experimental design of compartment-resolved intervention studies in tumor-bearing mice, including naïve controls where indicated, tumor-bearing controls, checkpoint-blockade groups, and pathway- or compartment-targeted intervention groups. **(B)** Echocardiographic assessment of cardiac function across experimental groups. **(C)** Circulating and tissue injury biomarkers across groups. **(D)** Flow-cytometric analysis of inflammatory effector populations. Axis labels and all experimental groups are shown for each plot. **(E)** Structural analysis of cardiomyocyte injury and membrane disruption. **(F)** Immunoblot or protein-quantification analysis of pyroptosis-related markers in myeloid-cell fractions and cardiomyocyte-enriched fractions. **(G)** Mitochondrial stress and oxidative injury markers in cardiomyocyte-enriched fractions. **(H)** Integrated relationship between cardiac injury indices and composite pyroptosis–mitochondrial scores across groups. In this figure, “myeloid blockade” refers to experimental reduction of myeloid inflammatory amplification, whereas the cardiomyocyte-directed intervention refers to attenuation of cardiomyocyte pyroptosis-related injury signaling rather than depletion of cardiomyocytes. The graded relationship in panel H indicates that increasing composite pyroptosis–mitochondrial scores are associated with progressively greater cardiac and systemic injury burden across groups. Statistical analyses were performed using one-way ANOVA or Kruskal–Wallis testing with appropriate multiple-comparison correction.

To define causal effector compartments, compartment-specific perturbation experiments were performed in a second intervention series. Myeloid blockade was achieved using anti-Ly6G antibody at 100 μg intraperitoneally every 3 days, starting 1 day before the first checkpoint-blockade dose. Cardiomyocyte-directed injury attenuation was modeled using the caspase-1 inhibitor VX-765 at 50 mg/kg intraperitoneally once daily, which was selected to suppress cardiomyocyte pyroptosis-related signaling rather than to deplete cardiomyocytes. This distinction was maintained throughout the manuscript to avoid the inaccurate implication that cardiomyocytes themselves were depleted. Myeloid and cardiomyocyte fractions were separately analyzed for pyroptosis-related proteins and mitochondrial stress markers. Myeloid fractions were isolated from splenic and cardiac CD11b-positive cells, whereas cardiomyocyte-enriched fractions were obtained from enzymatically dissociated ventricular tissue. These compartment-resolved experiments supported the interpretation of **Figures 2D–H** and the conclusion that both myeloid inflammatory amplification and cardiomyocyte stress contributed to the observed multi-organ injury patterns.

## 3 Results

### 3.1 Baseline characteristics

The baseline characteristics of the development cohort and the external validation cohort are summarized in **Table 1**. Overall, the two cohorts were broadly comparable across demographic, oncologic, treatment-related, and early critical care variables, supporting the use of the external dataset for independent validation rather than for derivation of the clinical phenotypes or prediction models.

**Table 1:** Baseline characteristics of critically ill cancer patients treated with PD-(L)1 and/or CTLA-4 inhibitors in the development and external validation cohorts.

Characteristic	Development cohort (n = 428)	External validation cohort (n = 176)	P value
Age, years, mean (SD)	63.7 (11.9)	62.8 (12.4)	0.396
Male, n (%)	268 (62.6)	112 (63.6)	0.826
Body mass index, kg/m², mean (SD)	24.1 (3.9)	23.8 (4.1)	0.438
Current smoker, n (%)	112 (26.2)	43 (24.4)	0.65
Cancer type, n (%)			
Lung cancer	168 (39.3)	72 (40.9)	0.706
Gastrointestinal cancer	86 (20.1)	33 (18.8)	0.72
Hepatobiliary/pancreatic cancer	44 (10.3)	17 (9.7)	0.822
Genitourinary cancer	48 (11.2)	20 (11.4)	0.95
Melanoma/skin cancer	22 (5.1)	10 (5.7)	0.746
Head and neck cancer	18 (4.2)	7 (4.0)	0.925
Other solid tumors	42 (9.8)	17 (9.7)	0.977
Stage IV or metastatic disease, n (%)	314 (73.4)	131 (74.4)	0.802
ECOG performance status ≥ 2, n (%)	236 (55.1)	101 (57.4)	0.605
Prior lines of systemic therapy, median (IQR)	2 (1–3)	2 (1–3)	0.913
ICI regimen, n (%)			
Anti–PD-(L)1 monotherapy	276 (64.5)	116 (65.9)	0.748
Anti–CTLA-4 monotherapy	31 (7.2)	12 (6.8)	0.865
Anti–PD-(L)1 + anti–CTLA-4	96 (22.4)	39 (22.2)	0.953
ICI + chemotherapy and/or targeted therapy	25 (5.8)	9 (5.1)	0.733
Time from last ICI dose to ICU admission, days, median (IQR)	12.6 (6.3–23.9)	13.4 (6.8–24.7)	0.521
History of irAEs before ICU admission, n (%)	84 (19.6)	37 (21.0)	0.692
Systemic corticosteroids within 7 days before ICU, n (%)	126 (29.4)	54 (30.7)	0.753
Comorbidities, n (%)			
Hypertension	214 (50.0)	86 (48.9)	0.81
Diabetes mellitus	118 (27.6)	45 (25.6)	0.617
Coronary artery disease	76 (17.8)	29 (16.5)	0.701
Chronic kidney disease (baseline eGFR < 60)	64 (15.0)	24 (13.6)	0.655
Chronic liver disease/cirrhosis	41 (9.6)	18 (10.2)	0.819
COPD/asthma	58 (13.6)	26 (14.8)	0.683
Prior autoimmune disease, n (%)	29 (6.8)	11 (6.3)	0.817
ICU admission profile			
Primary ICU admission category, n (%)			0.964
Acute respiratory failure	176 (41.1)	72 (40.9)	
Shock/hemodynamic instability	101 (23.6)	44 (25.0)	
Suspected irAE-related organ dysfunction	86 (20.1)	34 (19.3)	
Other (bleeding, neurologic, postoperative)	65 (15.2)	26 (14.8)	
APACHE II score, median (IQR)	18.4 (14.1–24.8)	18.9 (14.6–25.2)	0.611
SOFA score at ICU admission, median (IQR)	7.6 (5.0–10.9)	7.8 (5.1–11.2)	0.689
Suspected/confirmed infection at admission, n (%)	241 (56.3)	102 (58.0)	0.702
Positive blood culture within 48 h, n (%)	62 (14.5)	27 (15.3)	0.799
Organ support within first 24 h, n (%)			
Invasive mechanical ventilation	214 (50.0)	93 (52.8)	0.534
Non-invasive ventilation/HFNC	132 (30.8)	49 (27.8)	0.456
Vasopressors	196 (45.8)	84 (47.7)	0.671
Renal replacement therapy	71 (16.6)	31 (17.6)	0.772
Key laboratory values at ICU admission			
Lactate, mmol/L, median (IQR)	2.3 (1.5–3.9)	2.4 (1.6–4.1)	0.482
CRP, mg/L, median (IQR)	98.7 (44.2–167.9)	103.5 (47.6–172.4)	0.563
Procalcitonin, ng/mL, median (IQR)	0.86 (0.24–3.10)	0.93 (0.26–3.28)	0.412
D-dimer, mg/L FEU, median (IQR)	3.12 (1.46–6.58)	3.28 (1.51–6.74)	0.521
Platelets, ×10^9^/L, mean (SD)	173.6 (92.4)	169.8 (95.1)	0.647
Creatinine, µmol/L, median (IQR)	104.7 (78.9–156.3)	109.5 (80.6–162.8)	0.402
Total bilirubin, µmol/L, median (IQR)	18.9 (11.4–34.6)	19.6 (11.8–36.1)	0.593
ALT, U/L, median (IQR)	41.8 (22.5–86.7)	44.3 (23.1–90.4)	0.47
High-sensitivity troponin, ng/L, median (IQR)	38.6 (14.2–121.5)	41.1 (15.0–128.7)	0.538
PaO_2_/FiO_2_ ratio, mmHg, median (IQR)	186.4 (122.7–262.3)	181.9 (118.6–258.8)	0.513
Immune-toxicity–related phenotypes at/near admission, n (%)			
Suspected immune-related pneumonitis	74 (17.3)	32 (18.2)	0.784
Suspected immune-related myocarditis	21 (4.9)	9 (5.1)	0.926
Suspected immune-related hepatitis	33 (7.7)	14 (8.0)	0.901
Suspected immune-related nephritis	27 (6.3)	12 (6.8)	0.806

ICU, intensive care unit; ICI, immune checkpoint inhibitor; irAE, immune-related adverse event; ECOG, Eastern Cooperative Oncology Group; APACHE II, Acute Physiology and Chronic Health Evaluation II; SOFA, Sequential Organ Failure Assessment; HFNC, high-flow nasal cannula; CRP, C-reactive protein; ALT, alanine aminotransferase.

Continuous variables are presented as mean (SD) or median (IQR), as appropriate, and categorical variables are presented as n (%). P values compare the development and external validation cohorts and are provided to describe cohort comparability rather than to imply formal hypothesis testing of randomization-balanced groups.

The mean age was 63.7 years in the development cohort and 62.8 years in the external validation cohort, and both cohorts were predominantly male. Body mass index, smoking prevalence, performance status, and comorbidity profiles were also similar between cohorts. In both datasets, the tumor spectrum was dominated by lung cancer, followed by gastrointestinal, genitourinary, and hepatobiliary or pancreatic malignancies, and most patients had stage IV or metastatic disease, indicating a broadly comparable oncologic background.

Immune checkpoint inhibitor exposure patterns were likewise similar. Anti–PD-(L)1 monotherapy accounted for the largest proportion of treatment regimens in both cohorts, whereas anti–CTLA-4 monotherapy and combined anti–PD-(L)1 plus anti–CTLA-4 blockade represented smaller but clinically relevant subsets. The interval from the most recent ICI administration to ICU admission was comparable across cohorts, as were the prevalence of pre-ICU immune-related adverse events and short-term systemic corticosteroid exposure.

At ICU admission, the overall severity of illness was similar between cohorts. APACHE II and SOFA scores showed closely aligned distributions, and the leading admission categories were acute respiratory failure, shock or hemodynamic instability, and suspected immune-toxicity-associated organ dysfunction. Infection-related considerations were common in both cohorts, with similar frequencies of suspected or confirmed infection at admission and comparable blood-culture positivity rates within the first 48 h. Early organ-support requirements, including invasive mechanical ventilation, vasoactive support, and renal replacement therapy, were also well balanced between the two datasets.

Initial laboratory profiles showed no major between-cohort differences in lactate, inflammatory markers, coagulation indices, renal and hepatic function, cardiac injury biomarkers, or oxygenation status. Suspected immune-toxicity-related clinical events were identified in both cohorts, with pneumonitis representing the most frequent category, whereas myocarditis, hepatitis, and nephritis were less common. Taken together, these findings indicate that the development and external validation cohorts were clinically comparable at baseline, while remaining independent datasets suitable for downstream phenotype replication and model transportability assessment.

### 3.2 Co-occurrence network of immune toxicity and organ dysfunction

**Figure 3:**
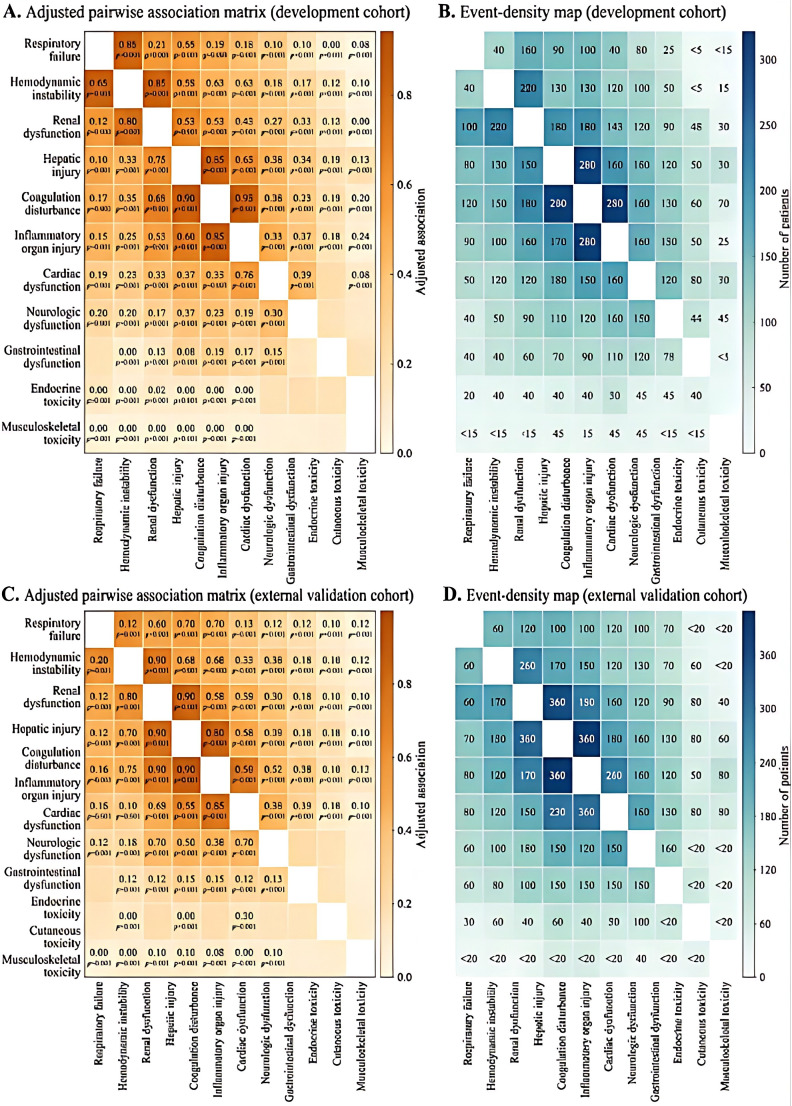
Pairwise co-occurrence networks of immune-toxicity events and organ dysfunction domains in critically ill cancer patients after recent PD-(L)1 and/or CTLA-4 inhibitor exposure. **(A)** Adjusted pairwise association matrix in the development cohort. Warmer colors indicate stronger positive co-occurrence between immune-toxicity events and organ dysfunction domains after accounting for baseline severity and major clinical covariates. **(B)** Event-density map corresponding to panel A, showing the number of patients contributing to each pairwise relationship in the development cohort. **(C)** Adjusted pairwise association matrix in the external validation cohort. **(D)** Event-density map corresponding to panel C in the external validation cohort. Pairwise associations were calculated using prespecified co-occurrence metrics as described in the Methods section. These panels are intended to visualize cross-organ connectivity and network reproducibility across cohorts rather than to imply direct causal relationships between any two individual domains.

To examine whether immune-toxicity-associated critical illness followed recognizable cross-organ architectures rather than isolated organ-specific events, we first constructed pairwise co-occurrence networks linking suspected immune-toxicity events and major organ dysfunction domains. As shown in **Figures 3A–D**, the development cohort demonstrated dense cross-domain connectivity, with respiratory, circulatory, renal, hepatic, coagulation, and myocardial injury domains showing frequent overlap. These findings indicate that organ injury after recent ICI exposure in critically ill patients rarely occurred in isolation and instead tended to cluster across physiologically related systems.

In the development cohort, the co-occurrence strength matrix (**Figure 3A**) showed that several organ domains were connected by moderate-to-strong pairwise associations after adjustment for baseline illness severity and major clinical confounders. The corresponding event-density map (**Figure 3B**) indicated that these associations were supported by substantial numbers of observed cases rather than by sparse or isolated combinations. The most connected regions of the network involved respiratory failure, hemodynamic instability, renal dysfunction, coagulation disturbance, and markers of inflammatory organ injury, supporting the concept of coordinated systemic deterioration rather than single-organ toxicity alone.

A similar overall architecture was observed in the external validation cohort. Although the absolute intensity of some pairwise associations differed modestly, the validation network again showed broad interconnection among respiratory, circulatory, renal, hepatic, and coagulation-related domains (**Figure 3C**), and the corresponding event-density map (**Figure 3D**) confirmed that the dominant network structure was reproduced in an independent dataset. Importantly, the validation cohort preserved the same high-connectivity backbone rather than generating an unrelated pattern of organ overlap.

Taken together, these analyses indicate that immune-toxicity-associated critical illness in ICI-exposed ICU patients is characterized by reproducible multi-organ coupling. This network-level structure provided the empirical basis for subsequent phenotype discovery, because it suggested that the observed clinical heterogeneity reflected organized cross-system patterns rather than random combinations of adverse events.

### 3.3 Data-driven MODS phenotypes and clinical interpretability

**Figure 4:**
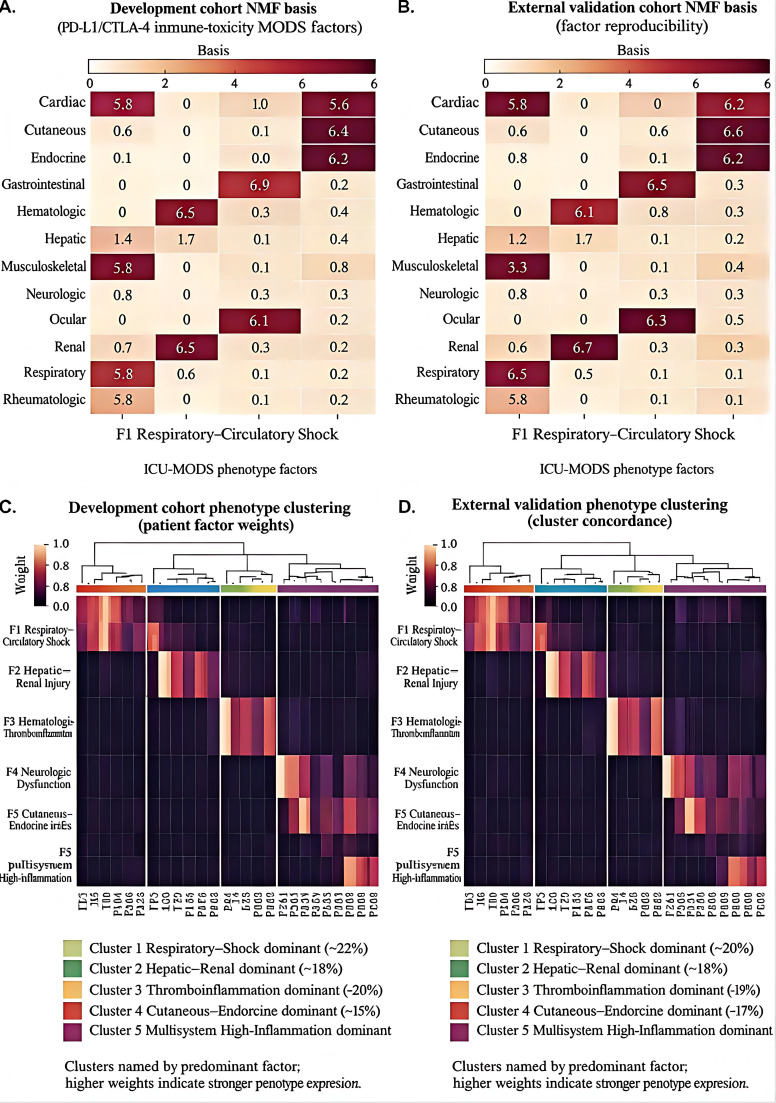
Data-driven phenotypes of immune-toxicity–associated MODS using NMF and hierarchical clustering. Data-driven phenotypes of immune-toxicity-associated MODS identified by non-negative matrix factorization and hierarchical clustering. **(A)** NMF loading matrix in the development cohort, showing the contribution of organ dysfunction, inflammatory, endothelial–coagulation, and organ-support variables to the latent factors. **(B)** NMF loading matrix in the external validation cohort. **(C)** Hierarchical clustering heatmap of patient-level factor weights in the development cohort, identifying four major phenotypes. **(D)** Hierarchical clustering heatmap of patient-level factor weights in the external validation cohort, showing reproduction of the same four dominant phenotype patterns with independent patient allocation. The four phenotypes were labeled according to their dominant clinical signatures: respiratory–circulatory dominant, hepatorenal injury dominant, hematologic–thromboinflammatory, and cutaneous–endocrine dominant. **(C, D)** are conceptually corresponding but are not duplicated; each panel represents independent clustering performed within its respective cohort.

Because the co-occurrence analyses suggested that organ injury was organized into reproducible cross-system patterns, we next performed data-driven phenotype discovery using non-negative matrix factorization followed by hierarchical clustering. As shown in **Figures 4A–D**, this approach identified four clinically interpretable phenotypes of immune-toxicity-associated MODS, and the overall phenotype structure was preserved in the external validation cohort.

In the development cohort, the NMF loading matrix (**Figure 4A**) revealed sparse and biologically interpretable factor structures, with each latent component weighted primarily toward a limited set of organ dysfunction and inflammatory domains rather than diffuse contributions from all variables. This pattern supported the presence of structured heterogeneity across critically ill ICI-exposed patients. In the external validation cohort, the factor-loading architecture (**Figure 4B**) remained directionally consistent, indicating that the dominant latent structure was not unique to the derivation sample.

At the patient level, hierarchical clustering of factor weights in the development cohort identified four major phenotypes: a respiratory–circulatory dominant phenotype, a hepatorenal injury dominant phenotype, a hematologic–thromboinflammatory phenotype, and a cutaneous–endocrine dominant phenotype with comparatively lower early multi-organ burden. These phenotype labels were assigned on the basis of their dominant clinical and biological feature patterns rather than on any single variable alone. The respiratory–circulatory phenotype was characterized by severe oxygenation impairment and hemodynamic instability, the hepatorenal phenotype by combined renal and hepatic dysfunction, the hematologic–thromboinflammatory phenotype by coagulation disturbance and inflammatory burden, and the cutaneous–endocrine phenotype by relatively lower immediate organ-support requirements together with more limited early systemic injury.

In the external validation cohort, hierarchical clustering yielded a phenotype configuration that reproduced the same four dominant patterns (**Figure 4D**). Although the exact ordering of individual patients and the relative compactness of the clusters were not identical to those in the development cohort, the main cross-domain phenotype structure remained stable. This distinction is important because the validation analysis was intended to test reproducibility of the clinical architecture rather than visual identity of the clustering heatmaps.

Overall, these findings indicate that immune-toxicity-associated MODS is not a single undifferentiated syndrome but instead comprises reproducible subgroups with distinct clinical signatures. This phenotypic structure formed the basis for the subsequent outcome comparisons and risk-stratification analyses.

### 3.4 Landmark outcomes across phenotypes

**Figure 5:**
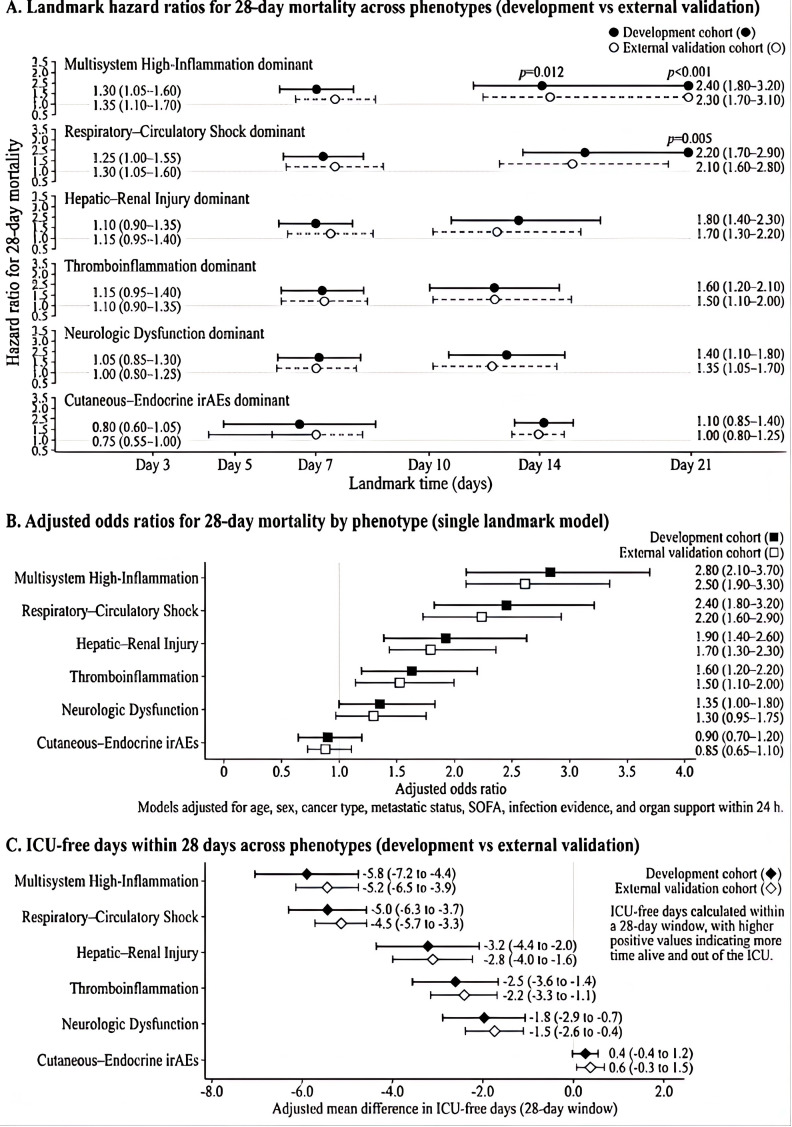
Landmark outcome comparisons across the four immune-toxicity-associated MODS phenotypes. **(A)** Adjusted hazard ratios for 28-day mortality across sequential landmark analyses. The cutaneous–endocrine dominant phenotype served as the reference group. Each dot represents the adjusted hazard ratio for a phenotype relative to the reference group, and the horizontal error bars indicate 95% confidence intervals. The P values represent overall between-phenotype comparisons at each landmark time point. **(B)** Adjusted effect estimates for 28-day mortality at the prespecified primary landmark time point. **(C)** Distribution of ICU-free days within 28 days across phenotypes. Models were adjusted for age, sex, metastatic disease, baseline SOFA score, evidence of infection, and early organ-support intensity. The figure is intended to show relative short-term outcome gradients across phenotypes rather than definitive causal effects of phenotype assignment.

Landmark analyses were performed to compare short-term outcomes across the four immune-toxicity-associated MODS phenotypes identified in the development cohort and replicated in the external validation cohort. As shown in **Figures 5A–C**, separation between phenotypes was already evident at the early landmark time points and remained directionally stable over time. Among the four phenotypes, the respiratory–circulatory dominant phenotype showed the highest 28-day mortality risk, followed by the hepatorenal injury dominant phenotype and the hematologic–thromboinflammatory phenotype, whereas the cutaneous–endocrine dominant phenotype consistently showed the lowest mortality risk.

In the time-updated landmark survival models (**Figure 5A**), hazard estimates for the respiratory–circulatory dominant phenotype remained highest across sequential landmark analyses. The cutaneous–endocrine dominant phenotype was used as the reference group because it showed the lowest early organ-support burden and the most favorable short-term trajectory. In **Figure 5A**, each dot represents the adjusted hazard ratio for 28-day mortality for a given phenotype relative to the cutaneous–endocrine reference group at the specified landmark time point, and the horizontal error bars indicate the corresponding 95% confidence intervals. The three global P values shown in the figure correspond to the overall between-phenotype comparison at each landmark time point.

Single-landmark adjusted analyses at the prespecified evaluation window yielded similar results (**Figure 5B**). Relative to the cutaneous–endocrine dominant phenotype, the respiratory–circulatory dominant phenotype retained the largest adverse effect estimate, whereas the hepatorenal injury dominant and hematologic–thromboinflammatory phenotypes occupied intermediate positions. The same ordering was observed for ICU-free days within 28 days (**Figure 5C**), with the respiratory–circulatory phenotype showing the lowest ICU-free days and the cutaneous–endocrine phenotype showing the most favorable distribution.

These outcome gradients were consistent with the biological and clinical profiles of the four phenotypes. Patients in the respiratory–circulatory dominant phenotype had the greatest burden of hypoxemia, hemodynamic instability, and early organ-support use, whereas patients in the hepatorenal injury dominant phenotype showed a higher requirement for renal replacement therapy and a prolonged recovery trajectory. The hematologic–thromboinflammatory phenotype showed intermediate mortality but a substantial burden of vasopressor use and coagulation-related dysfunction. By contrast, the cutaneous–endocrine dominant phenotype had comparatively limited early multi-organ burden and the most favorable short-term course. Similar directional trends were observed in the external validation cohort, supporting the reproducibility of the phenotype–outcome relationship across datasets.

Differences across phenotypes were further quantified from an endpoint perspective in **Table 2**. The 28-day mortality rate demonstrated a graded distribution among phenotypic groups, with high-risk subtypes retaining significant relative effect sizes after multivariable adjustment. At the level of organ support, high-risk phenotypes showed higher frequencies of invasive mechanical ventilation and vasoactive therapy. The requirement for renal replacement therapy was also more common in the hepatic–renal dominant and multisystem high-inflammation phenotypes. These findings describe consistent variation in outcome burden across phenotypic strata.

**Table 2:** Landmark outcome comparison across immune-toxicity–associated MODS phenotypes: 28-day mortality, organ support, and ICU length of stay.

Phenotype (predominant factor)	n	28-day mortality, n (%)	Adjusted OR for 28-day mortality (95% CI)	Invasive mechanical ventilation, n (%)	Vasopressor use, n (%)	Renal replacement therapy, n (%)	ICU length of stay, days, median (IQR)	ICU-free days (0–28), median (IQR)
Cutaneous–Endocrine dominant	86	13 (15.1)	1.00 (reference)	29 (33.7)	28 (32.6)	8 (9.3)	6.1 (3.7–10.4)	20.1 (13.2–24.0)
Hematologic–Thromboinflammatory dominant	104	24 (23.1)	1.64 (0.82–3.26)	53 (51.0)	56 (53.8)	19 (18.3)	8.8 (5.1–14.9)	15.2 (7.6–21.3)
Hepatorenal Injury dominant	98	29 (29.6)	2.08 (1.05–4.12)	57 (58.2)	60 (61.2)	31 (31.6)	10.9 (6.3–18.1)	11.4 (4.2–18.0)
Respiratory–Circulatory dominant	140	50 (35.7)	2.91 (1.52–5.57)	108 (77.1)	111 (79.3)	30 (21.4)	13.2 (7.8–21.4)	7.9 (0.0–15.8)
P value overall		0.001		<0.001	<0.001	0.002	0.004	<0.001

Adjusted odds ratios were derived from multivariable logistic regression using the cutaneous–endocrine dominant phenotype as the reference group. Models were adjusted for age, sex, metastatic disease, baseline SOFA score, evidence of infection, and early organ-support intensity.

### 3.5 Predictors of MODS and robust feature selection

Candidate predictors of immune-toxicity-associated MODS within 72 h were evaluated using both conventional multivariable regression and penalized modeling approaches. The results are summarized in **Table 3**. Overall, markers of early physiologic severity, organ-support dependence, inflammatory–coagulation disturbance, and hepatorenal injury showed the most stable contribution across modeling strategies.

**Table 3:** Independent predictors of immune-toxicity-associated MODS within 72 h in critically ill ICU cancer patients.

Candidate predictor	Univariable OR (95% CI)	P value	Multivariable adjusted OR (95% CI)	P value	Penalized Model selection (LASSO/Elastic Net)	Standardized coefficient (β)
Age (per 10-year increase)	1.18 (1.04–1.35)	0.012	1.12 (0.97–1.30)	0.118	Not retained/Not retained	0.04
Male sex	1.09 (0.78–1.53)	0.616	1.06 (0.73–1.54)	0.754	Not retained/Not retained	0.02
Metastatic disease (yes)	1.42 (1.01–2.01)	0.044	1.28 (0.87–1.88)	0.212	Not retained/Retained	0.07
Anti–PD-(L)1 + anti–CTLA-4 (vs monotherapy)	1.76 (1.18–2.63)	0.006	1.58 (1.02–2.46)	0.040	Retained/Retained	0.12
Time from last ICI dose to ICU admission (per 7 days)	0.93 (0.86–0.99)	0.028	0.95 (0.88–1.02)	0.166	Not retained/Not retained	-0.03
Evidence of infection at admission (suspected/confirmed)	1.84 (1.31–2.60)	<0.001	1.62 (1.09–2.41)	0.018	Retained/Retained	0.15
SOFA score (per 1-point increase)	1.29 (1.20–1.39)	<0.001	1.21 (1.11–1.32)	<0.001	Retained/Retained	0.26
APACHE II score (per 5-point increase)	1.33 (1.18–1.49)	<0.001	1.10 (0.96–1.27)	0.170	Not retained/Not retained	0.05
Lactate, mmol/L (per 1 mmol/L)	1.22 (1.12–1.34)	<0.001	1.14 (1.03–1.27)	0.011	Retained/Retained	0.13
CRP, mg/L (per 50 mg/L)	1.17 (1.05–1.31)	0.005	1.09 (0.96–1.24)	0.180	Not retained/Retained	0.06
Procalcitonin, ng/mL (log-transformed)	1.28 (1.10–1.50)	0.002	1.12 (0.94–1.33)	0.204	Not retained/Not retained	0.04
D-dimer, mg/L FEU (per 1 mg/L)	1.09 (1.04–1.15)	<0.001	1.06 (1.01–1.12)	0.019	Retained/Retained	0.10
hs-Troponin, ng/L (per 50 ng/L)	1.11 (1.03–1.20)	0.007	1.07 (0.98–1.17)	0.136	Not retained/Retained	0.05
ALT, U/L (per 50 U/L)	1.14 (1.04–1.25)	0.006	1.10 (0.99–1.23)	0.079	Not retained/Retained	0.05
Total bilirubin, µmol/L per10µmol/L	1.23 (1.12–1.36)	<0.001	1.18 (1.06–1.33)	0.004	Retained/Retained	0.14
Creatinine, µmol/L per50µmol/L	1.21 (1.09–1.34)	<0.001	1.16 (1.03–1.31)	0.014	Retained/Retained	0.11
Platelets, ×10^9^/L (per 50 decrease)	1.19 (1.06–1.34)	0.004	1.12 (0.98–1.29)	0.094	Not retained/Retained	0.05
PaO_2_/FiO_2_ (per 50 mmHg decrease)	1.27 (1.14–1.43)	<0.001	1.18 (1.03–1.35)	0.015	Retained/Retained	0.12
Invasive mechanical ventilation within 24 h	2.34 (1.64–3.33)	<0.001	1.71 (1.12–2.61)	0.013	Retained/Retained	0.16
Vasopressor use within 24 h	2.58 (1.82–3.66)	<0.001	1.86 (1.22–2.83)	0.004	Retained/Retained	0.18
Baseline chronic kidney disease (eGFR < 60)	1.46 (0.98–2.19)	0.062	1.22 (0.78–1.92)	0.383	Not retained/Not retained	0.03
Prior autoimmune disease	1.33 (0.74–2.39)	0.344	1.19 (0.61–2.31)	0.610	Not retained/Not retained	0.02

Among severity-related variables, higher SOFA score was the most consistently retained predictor in both standard and penalized models. Early dependence on invasive mechanical ventilation and vasopressor support also remained strongly associated with subsequent MODS, indicating that early cardiopulmonary instability captured an important component of short-term deterioration risk. Markers of impaired perfusion and oxygenation, particularly elevated lactate and lower PaO_2_/FiO_2_ ratio, were also independently associated with increased MODS risk.

Inflammation- and coagulation-related variables contributed additional prognostic information. Evidence of infection at admission was retained in both the multivariable and penalized models, reflecting the clinical complexity of critically ill patients with overlapping inflammatory syndromes. D-dimer also showed robust selection across modeling strategies, supporting the relevance of thromboinflammatory activation. With respect to organ-specific injury, total bilirubin and creatinine were independently retained, indicating that early hepatorenal dysfunction contributed meaningfully to the development of full MODS.

Treatment-related exposure variables showed a more selective pattern. Combination anti–PD-(L)1 plus anti–CTLA-4 therapy remained independently associated with increased MODS risk after adjustment, whereas age, sex, and baseline chronic kidney disease did not show stable retention. Taken together, these findings indicate that early MODS risk in ICI-exposed critically ill patients is shaped by a combination of baseline severity, acute organ-support burden, thromboinflammatory activation, hepatorenal injury, and intensified checkpoint blockade exposure.

### 3.6 Model performance, incremental value, and calibration

The predictive performance of the baseline severity model and the augmented biomarker-informed models is summarized in **Table 4**. Overall, the baseline model based on conventional severity scores showed stable discrimination in both the development and external validation cohorts. However, the addition of immune-toxicity-related biomarker domains, including myocardial injury, coagulation activation, hepatorenal injury, and inflammatory burden, consistently improved model performance across datasets.

**Table 4:** Incremental predictive value of immune-toxicity-related biomarkers beyond clinical severity scores for MODS prediction.

Cohort	Model specification	AUC (95% CI)	ΔAUC vs base	Brier score	Calibration slope	Calibration-in-the-large	NRI (events/non-events)	IDI	Net benefit gain at Pt = 0.30
Development	Base clinical score model (SOFA + APACHE II)	0.781 (0.744–0.818)	Reference	0.178	0.92	0.03	Reference	Reference	Reference
Development	Base + hs-troponin + D-dimer	0.812 (0.778–0.846)	0.031	0.168	0.97	0.02	0.184 0.102/0.082	0.026	0.018
Development	Base + bilirubin + creatinine	0.821 (0.789–0.853)	0.040	0.165	1.01	0.01	0.203 0.116/0.087	0.031	0.022
Development	Base + CRP + procalcitonin	0.808 (0.773–0.843)	0.027	0.170	0.95	0.02	0.171 0.098/0.073	0.022	0.016
Development	Full biomarker panel (troponin, D-dimer, bilirubin, creatinine, CRP)	0.844 (0.813–0.875)	0.063	0.155	1.04	0.00	0.286 0.168/0.118	0.046	0.035
External validation	Base clinical score model (SOFA + APACHE II)	0.762 (0.706–0.818)	Reference	0.189	0.88	0.05	Reference	Reference	Reference
External validation	Base + hs-troponin + D-dimer	0.793 (0.742–0.844)	0.031	0.180	0.93	0.03	0.162 0.091/0.071	0.021	0.014
External validation	Base + bilirubin + creatinine	0.801 (0.751–0.851)	0.039	0.176	0.96	0.02	0.181 0.104/0.077	0.026	0.017
External validation	Base + CRP + procalcitonin	0.786 (0.734–0.838)	0.024	0.183	0.91	0.03	0.148 0.083/0.065	0.018	0.012
External validation	Full biomarker panel (troponin, D-dimer, bilirubin, creatinine, CRP)	0.822 (0.775–0.869)	0.060						

The base model included SOFA and APACHE II. Biomarker augmentation was evaluated incrementally to assess added prognostic value beyond conventional severity scores. NRI indicates net reclassification improvement, and IDI indicates integrated discrimination improvement.

In the development cohort, sequential addition of biomarker panels produced progressive gains in discrimination, with the full biomarker model achieving the highest AUC and the lowest Brier score. A similar pattern was observed in the external validation cohort, indicating that the incremental value of these biomarkers was not confined to the derivation sample. Importantly, the direction and magnitude of improvement were broadly preserved after external validation, supporting the transportability of the augmented risk framework.

Beyond discrimination, the augmented models also improved patient classification. Compared with the baseline model, the full biomarker panel yielded higher net reclassification improvement and integrated discrimination improvement in both cohorts, indicating better separation between higher-risk and lower-risk patients. These gains were particularly relevant near clinically meaningful decision thresholds, where more accurate early stratification may influence monitoring intensity, multidisciplinary review, and organ-support planning.

Calibration performance also improved after biomarker augmentation. The calibration slope and calibration-in-the-large values of the full model were closer to the ideal values than those of the baseline model, suggesting better alignment between predicted and observed MODS risk. Decision-curve analysis further indicated greater net benefit across typical threshold probabilities, supporting the potential clinical usefulness of the augmented model for early risk assessment in critically ill patients after recent checkpoint inhibitor exposure.

Taken together, these findings show that conventional severity scores capture an important part of early MODS risk, but biomarker-informed augmentation provides additional clinically meaningful information. This result is consistent with the broader framework of the study, in which phenotypic heterogeneity and biologically supported injury pathways jointly contribute to more refined risk stratification.

### 3.7 Clinical utility and risk-stratified pathway outputs

To translate model-derived probabilities into a clinically usable framework, predicted MODS risk was stratified into four operational categories, each linked to a corresponding alert threshold and escalation pathway (**Table 5**). Across both the development and external validation cohorts, outcome burden increased in a graded fashion from the low-risk to the very-high-risk tier. Specifically, the incidence of MODS within 72 h, 28-day mortality, and the need for major organ-support interventions rose progressively across categories, whereas ICU-free days declined accordingly. This pattern indicates that the model outputs were not only statistically discriminative but also clinically stratifiable.

**Table 5:** Clinical utility of the MODS risk model: operational risk groups, alert thresholds, pathway outputs, and associated outcome gradients.

Item	Low risk	Intermediate risk	High risk	Very high risk
Predicted MODS risk range	<0.12	0.12–0.24	0.24–0.40	>0.40
Recommended alert threshold (action trigger)	No alert	Soft alert if ≥0.20	Hard alert if ≥0.30	Critical alert if ≥0.45
Typical pathway output (risk-stratified bundle)	Standard ICU care; routine irAE screen; daily SOFA reassessment	Intensified monitoring every 6–8 h; repeat lactate, D-dimer, troponin; targeted imaging if clinical decline	Multidisciplinary review (ICU, oncology, infectious disease); structured immune-toxicity adjudication; proactive organ-support readiness	Immediate escalation; frequent respiratory and hemodynamic reassessment; expedited etiologic clarification; early readiness for ventilation, vasopressors, or renal support
Development cohort: patients, n (%)	126 (29.4)	132 (30.8)	103 (24.1)	67 (15.7)
Development cohort: MODS within 72 h, n (%)	9 (7.1)	23 (17.4)	39 (37.9)	39 (58.2)
Development cohort: 28-day mortality, n (%)	12 (9.5)	22 (16.7)	32 (31.1)	31 (46.3)
Development cohort: invasive ventilation, n (%)	31 (24.6)	54 (40.9)	63 (61.2)	55 (82.1)
Development cohort: vasopressors, n (%)	29 (23.0)	55 (41.7)	65 (63.1)	57 (85.1)
Development cohort: RRT, n (%)	7 (5.6)	15 (11.4)	23 (22.3)	24 (35.8)
Development cohort: ICU LOS, days, median (IQR)	5.1 (3.1–8.4)	7.6 (4.6–12.3)	10.9 (6.7–17.8)	14.2 (8.4–23.6)
Development cohort: ICU-free days (0–28), median (IQR)	21.8 (17.3–24.5)	17.2 (10.4–22.0)	10.6 (2.8–17.1)	5.4 (0.0–12.7)
External validation: patients, n (%)	49 (27.8)	54 (30.7)	45 (25.6)	28 (15.9)
External validation: MODS within 72 h, n (%)	4 (8.2)	11 (20.4)	18 (40.0)	16 (57.1)
External validation: 28-day mortality, n (%)	5 (10.2)	10 (18.5)	15 (33.3)	13 (46.4)
External validation: ICU LOS, days, median (IQR)	5.4 (3.2–9.0)	7.9 (4.7–12.8)	11.4 (6.9–18.4)	14.8 (8.9–24.2)

Risk categories were derived from the calibrated full model. These strata are intended to support structured escalation of monitoring and multidisciplinary assessment. They should not be interpreted as a definitive diagnostic classification of immune toxicity versus infection or other competing causes of critical illness.

In the development cohort, patients classified as low risk showed a relatively low short-term event burden, whereas those in the high-risk and very-high-risk categories had substantially higher rates of MODS, invasive ventilation, vasopressor use, and renal replacement therapy. The same directional pattern was observed in the external validation cohort, supporting the transportability of the risk-stratification framework beyond the derivation sample. Importantly, the differences across strata were not restricted to the primary endpoint alone but extended to broader indicators of ICU resource utilization and recovery trajectory, including ICU length of stay and ICU-free days.

At the clinical pathway level, each risk category was linked to a stepwise escalation bundle rather than to an automated treatment decision. The low-risk tier corresponded to standard ICU care with routine surveillance for possible immune-related toxicity. The intermediate-risk tier triggered intensified physiologic monitoring and repeat assessment of key laboratory markers. The high-risk tier prompted multidisciplinary review, structured adjudication of immune-toxicity-associated injury versus competing causes of deterioration, and preparation for organ-support escalation. The very-high-risk tier identified patients requiring immediate reassessment, rapid clarification of competing etiologies, and early readiness for respiratory, hemodynamic, or renal support. This framework was designed to support structured clinical response and earlier risk-informed evaluation, not to serve as a stand-alone diagnostic tool distinguishing immune toxicity from infection or other inflammatory conditions.

### 3.8 Mechanistic evidence supporting immune-toxicity-associated MODS

To determine whether the clinical phenotypes and risk-model signals were supported by biologically coherent injury programs, we integrated findings from serial patient biospecimens, *in vitro* plasma-transfer experiments, and *in vivo* checkpoint-blockade models. Across these complementary layers, the data consistently supported prominent activation of pyroptosis-related signaling, myeloid inflammatory amplification, endothelial–coagulation coupling, and mitochondrial stress in patients and models with immune-toxicity-associated MODS. These analyses were designed to identify dominant and experimentally actionable mechanisms rather than to claim that pyroptosis was the sole initiating pathway.

**Figure 6:**
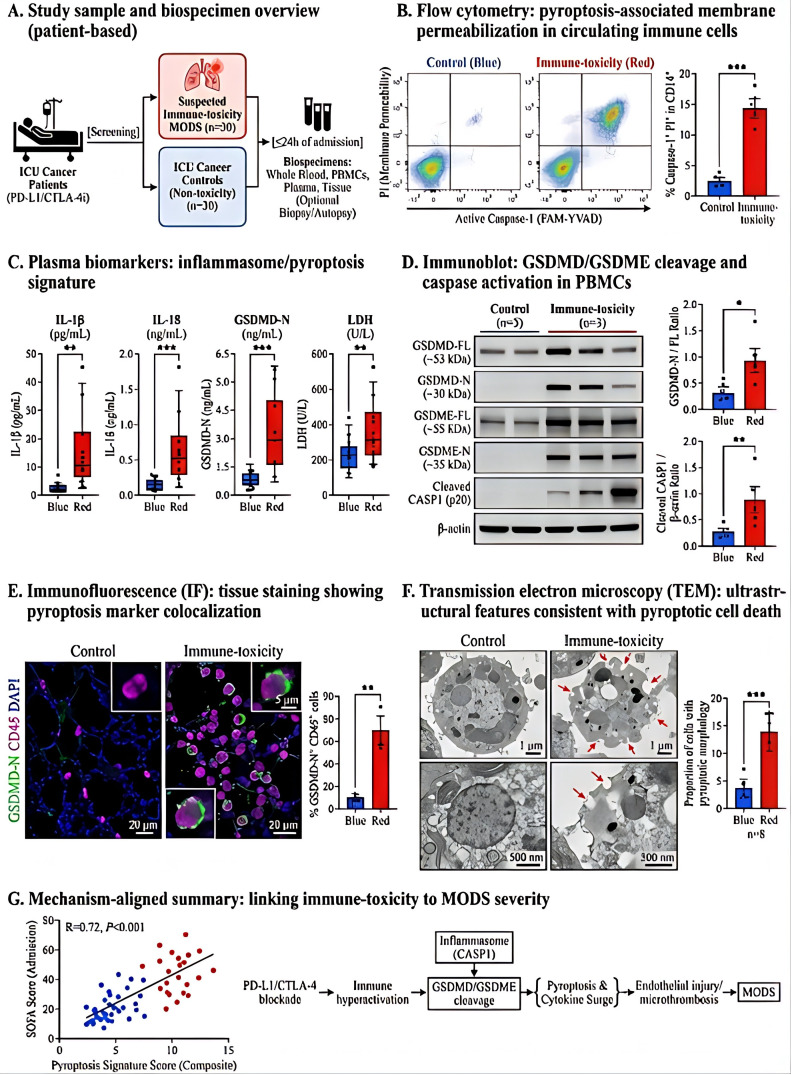
Evidence of pyroptosis-related immune-cell injury in ICU patients with suspected PD-(L)1/CTLA-4 inhibitor-associated immune toxicity. **(A)** Study workflow for serial biospecimen collection and downstream analyses in the human mechanistic cohort. **(B)** Flow-cytometric assessment of membrane permeabilization and active caspase-1 in circulating immune-cell populations. **(C)** Plasma concentrations of inflammasome- and pyroptosis-related mediators. **(D)** Immunoblot analysis of PBMC lysates showing full-length and cleaved pyroptosis-related proteins, including caspase-1, GSDMD, and GSDME. PBMCs were used for immunoblotting in this panel. **(E)** Immunofluorescence staining showing localization of pyroptosis-related markers within immune-cell compartments. **(F)** Representative transmission electron microscopy images showing membrane rupture, swelling, and ultrastructural injury compatible with inflammatory cell death. **(G)** Correlation between composite pyroptosis score and organ dysfunction severity indices. Data are shown as individual values with summary statistics as indicated in the figure panels. Between-group comparisons were performed using t tests, Mann–Whitney U tests, one-way ANOVA, or Kruskal–Wallis tests as appropriate. Correlations were assessed using Spearman analysis. The figure demonstrates prominent pyroptosis-related activation but does not exclude the contribution of other regulated cell-death pathways. The symbols indicate: *P < 0.05, **P < 0.01, and ***P < 0.001.

In the human biospecimen cohort, serial sampling revealed convergent evidence of inflammatory cell-death activation (**Figures 6A–G**). Flow cytometry demonstrated increased membrane permeabilization together with elevated active caspase-1 signals in circulating immune cells from patients with immune-toxicity-associated MODS compared with lower-burden or improving cases (**Figure 6B**). Plasma measurements showed corresponding increases in inflammasome- and pyroptosis-related mediators, including IL-1β, IL-18, HMGB1, and other inflammatory danger signals (**Figure 6C**). Immunoblot analysis of PBMC lysates further demonstrated increased cleavage of GSDMD and GSDME together with activation of caspase pathways (**Figure 6D**). These molecular changes were supported by immunofluorescence, which demonstrated increased cellular localization of pyroptosis-related markers within immune-cell compartments (**Figure 6E**), and by transmission electron microscopy, which revealed membrane rupture, cytoplasmic swelling, and organelle disruption compatible with pyroptotic injury (**Figure 6F**). When integrated into a composite score, the pyroptosis-related signature correlated positively with increasing SOFA burden and multi-organ dysfunction severity (**Figure 6G**), indicating that inflammatory cell-death activation tracked with clinical deterioration.

Based on these human observations, we next tested whether pathway-directed intervention could attenuate organ injury in preclinical models (**Figures 1A–H**). In tumor-bearing mice exposed to anti–PD-(L)1 and/or anti–CTLA-4 treatment, checkpoint blockade was associated with increased biochemical evidence of organ injury, more severe histopathologic damage, and broader inflammatory infiltration across the heart, lung, liver, and kidney. Compared with ICI treatment alone, pharmacologic inhibition of pyroptosis-related signaling or mitochondrial oxidative stress reduced circulating and tissue injury readouts (**Figure 1B**), improved semiquantitative histopathology scores (**Figure 1C**), and decreased immune-cell infiltration on immunohistochemistry and immunofluorescence (**Figure 1D**). Immunoblot analysis showed reduced cleavage of caspase-1, GSDMD, and GSDME after intervention (**Figure 1E**), while mitochondrial stress markers, including loss of membrane potential, oxidative injury, and structural disruption, were correspondingly attenuated (**Figure 1F**). Tissue architecture, including cardiomyocyte integrity, was also partially preserved under pathway-directed intervention (**Figure 1G**), and ultrastructural analyses further confirmed mitigation of membrane and mitochondrial injury morphology (**Figure 1H**). Together, these results support a functional contribution of pyroptosis-related and mitochondrial injury pathways to checkpoint-blockade-associated systemic damage.

**Figure 7:**
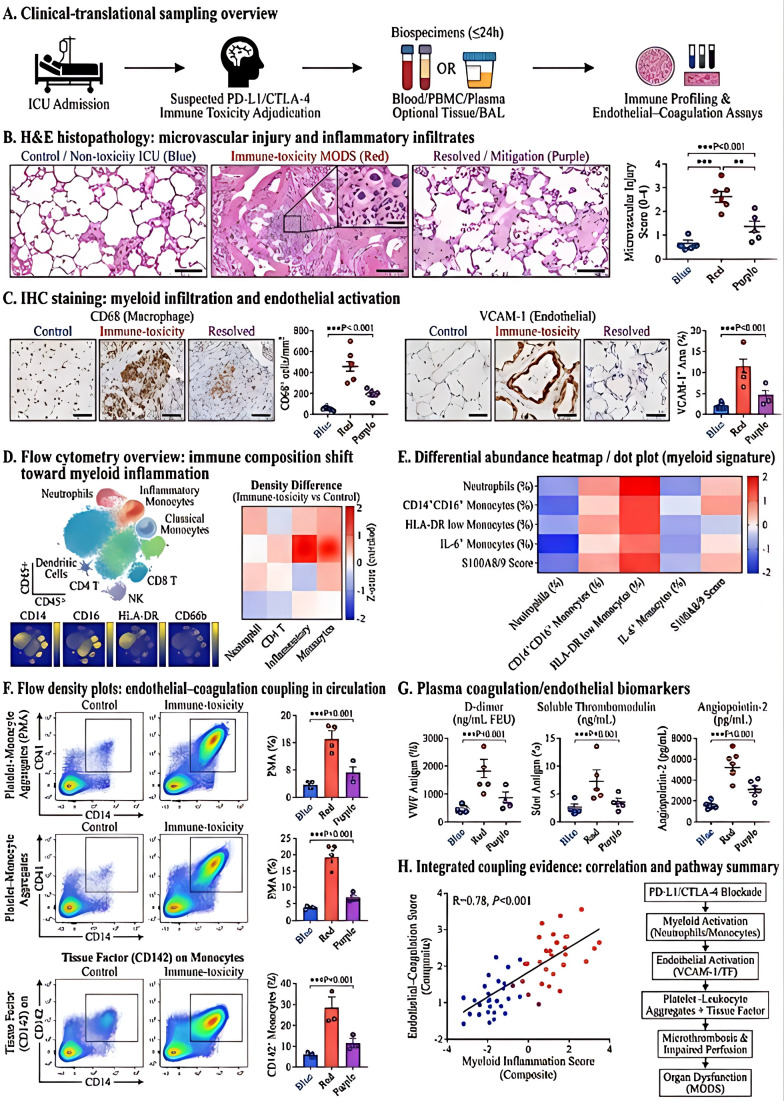
Myeloid-dominated inflammation and endothelial–coagulation coupling in immune-toxicity-associated MODS. **(A)** Clinical sampling and analytic framework. **(B)** Histopathology showing tissue injury and microvascular disruption across comparison groups. **(C)** Immunohistochemical assessment of myeloid infiltration and endothelial activation. **(D)** Flow-cytometric profiling of circulating immune composition. **(E)** Relative abundance of inflammatory monocyte and neutrophil-associated signatures. **(F)** Platelet–leukocyte aggregates and tissue factor-related measurements. **(G)** Plasma markers of endothelial perturbation and coagulation activation. **(H)** Correlation analysis linking myeloid inflammation scores with endothelial–coagulation indices. Group comparisons were performed using parametric or nonparametric tests according to distribution. Correlation analysis was performed using Spearman coefficients. These panels support coupling between innate immune amplification and vascular injury rather than implying a single unidirectional causal pathway. The symbols indicate: *P < 0.05, **P < 0.01, and ***P < 0.001.

We then examined how these mechanisms were linked to the inflammatory microenvironment and vascular injury programs observed in the clinical setting (**Figures 7A–H**). Histopathology demonstrated increased microvascular injury, interstitial inflammatory infiltration, and tissue-level structural disruption in patients with immune-toxicity-associated MODS relative to lower-burden or clinically improving cases (**Figure 7B**). Immunohistochemistry further showed increased myeloid-cell accumulation and endothelial activation, both of which were less pronounced in the clinically mitigated group (**Figure 7C**). Flow-cytometric profiling revealed a shift toward myeloid predominance in the peripheral immune compartment (**Figure 7D**), with concordant increases in inflammatory monocyte and neutrophil-associated signatures (**Figure 7E**). In parallel, platelet–leukocyte aggregates, tissue factor expression, and circulating markers of endothelial perturbation and coagulation activation were increased in the high-burden group and decreased in the mitigated group (**Figures 7F, G**). Correlation analysis showed that higher myeloid inflammation scores tracked closely with endothelial–coagulation indices (**Figure 7H**), supporting the concept that innate immune amplification and vascular injury were biologically coupled rather than independent processes.

Finally, compartment-resolved analyses were used to clarify which cellular compartments contributed most directly to the observed injury phenotype (**Figures 2A–H**). In tumor-bearing mice, checkpoint blockade impaired cardiac function on echocardiography, whereas pathway-targeted intervention and compartment-specific modulation led to partial functional recovery (**Figure 2B**). Circulating and tissue injury biomarkers followed the same directional pattern (**Figure 2C**). Flow-cytometric analysis demonstrated redistribution of inflammatory effector populations across experimental groups (**Figure 2D**), indicating that the injury phenotype was accompanied by changes in immune composition rather than by isolated parenchymal damage alone. Cardiomyocyte structural analyses showed differential injury morphology and membrane disruption across treatment conditions (**Figure 2E**). At the molecular level, pyroptosis-related proteins were activated in both myeloid-cell fractions and cardiomyocyte-enriched fractions, although the magnitude and pattern of activation differed by compartment (**Figure 2F**). Mitochondrial stress markers in cardiomyocytes, including oxidative injury and loss of structural integrity, were also modulated by targeted intervention (**Figure 2G**). The integrated analysis shown in **Figure 2H** demonstrated a graded relationship, meaning that higher composite pyroptosis–mitochondrial scores were associated with progressively greater cardiac injury and broader multi-organ burden across experimental groups, rather than merely a binary difference between treated and untreated animals. This result supports a dose-like biological gradient linking inflammatory cell-death activation, cardiomyocyte stress, and systemic injury severity.

Taken together, the mechanistic data provide biological support for the clinical framework identified in the earlier sections of the study. Specifically, they indicate that immune-toxicity-associated MODS is accompanied by coordinated activation of pyroptosis-related signaling, myeloid inflammatory amplification, endothelial–coagulation perturbation, and mitochondrial injury. At the same time, these experiments were not designed to exclude other regulated cell-death programs, such as necroptosis or ferroptosis. The present findings therefore support pyroptosis as a prominent and experimentally actionable pathway within a broader multi-mechanism injury network.

## 4 Discussion

This study provides a translational framework for understanding immune-toxicity-associated critical illness in tumor patients recently exposed to PD-(L)1 and/or CTLA-4 blockade. The main finding is that organ injury in this setting does not behave as a collection of isolated single-organ adverse events. Instead, in critically ill patients, immune-toxicity-associated deterioration is organized as a cross-system process involving recurrent patterns of respiratory, circulatory, hepatorenal, thromboinflammatory, and endocrine-associated injury. This systems-level organization helps explain why patients with apparently similar oncologic backgrounds may nevertheless follow markedly different ICU trajectories, with substantial variation in short-term mortality, organ-support requirements, and recovery potential.

A first important contribution of the study is the demonstration that immune-toxicity-associated organ dysfunction shows a reproducible co-occurrence architecture across clinical cohorts. The observed linkages among respiratory failure, hemodynamic instability, coagulation disturbance, renal injury, and other domains were not consistent with random superposition alone. Rather, they suggest that severe checkpoint-inhibitor-associated toxicity in the ICU often evolves through shared upstream biological programs that affect multiple organ systems simultaneously. On this basis, the data-driven phenotype analysis further resolved this heterogeneity into a limited number of clinically interpretable subgroups with distinct short-term outcome gradients. This is clinically relevant because it shifts the conceptual focus from isolated organ-specific irAEs toward a stratifiable syndrome of systemic injury, which is more aligned with the way critically ill patients actually present in practice (11, 12).

A second key finding is that phenotype-based heterogeneity was not merely descriptive but was linked to meaningful differences in mortality, organ-support burden, and ICU resource use. The respiratory–circulatory dominant phenotype showed the highest short-term risk and the lowest ICU-free days, whereas the cutaneous–endocrine dominant phenotype showed the most favorable early trajectory. The hepatorenal injury dominant and hematologic–thromboinflammatory phenotypes occupied intermediate positions, but with distinct patterns of renal replacement therapy, vasopressor use, and inflammatory burden. These gradients support the argument that early critical deterioration after checkpoint blockade is not clinically uniform and that conventional severity scoring alone may not fully capture its structure. The incremental improvement observed after incorporating immune-toxicity-related biomarker domains is consistent with this interpretation. Rather than replacing standard ICU assessment, these biomarker-informed models appear to add complementary information that may help organize surveillance intensity, multidisciplinary review, and timing of escalation (13, 14).

At the same time, the present findings should not be interpreted as establishing a definitive diagnostic tool for distinguishing immune-toxicity-associated organ injury from infection or other causes of critical illness. This point is important. In real ICU settings, patients often present with overlapping inflammatory syndromes, including infection, tumor progression, prior treatment injury, and noninfectious hyperinflammatory states. Although the current framework may support earlier recognition, more structured adjudication, and risk-informed clinical assessment, it was not designed as an infection-controlled diagnostic study. Therefore, the results are better understood as providing a biologically informed stratification framework than as proving diagnostic separation between competing etiologies. This limitation directly reflects the complexity of ICU oncology practice and should frame how the model is interpreted and applied.

Mechanistically, the study supports a model in which inflammatory cell-death signaling, myeloid inflammatory amplification, endothelial activation, coagulation perturbation, and mitochondrial stress act in concert during progression toward multi-organ dysfunction. Among these mechanisms, pyroptosis emerged as a prominent and experimentally tractable pathway. In the human biospecimen cohort, increased membrane permeabilization, active caspase-1 signaling, cleavage of GSDMD and GSDME, and elevation of pyroptosis-related circulating mediators all supported enhanced inflammatory cell-death activity in patients with higher organ-dysfunction burden. These findings were reinforced by ultrastructural observations and by the positive relationship between composite pyroptosis signatures and severity indices. Taken together, these data indicate that pyroptosis-related activation is not an isolated molecular signal but part of a broader injury program associated with clinically relevant deterioration (15).

The present results also extend this interpretation by linking pyroptosis-related activation to myeloid-dominated inflammation and endothelial–coagulation coupling. Increased inflammatory monocyte and neutrophil signatures, together with higher levels of platelet–leukocyte aggregates, tissue factor expression, and endothelial perturbation markers, suggest that innate immune amplification and vascular injury are biologically connected. One plausible interpretation is that pyroptotic signaling amplifies release of danger-associated mediators and cytokines, which in turn sustain myeloid recruitment, endothelial activation, microvascular dysfunction, and coagulation imbalance. Through this pathway, injury that may begin in one compartment can propagate across vascular and metabolic systems and become clinically manifest as multi-organ dysfunction rather than as localized toxicity alone (16).

Mitochondrial dysfunction appears to be an important part of this escalation process rather than a secondary epiphenomenon. In both *in vitro* and *in vivo* experiments, checkpoint-blockade-associated injury was accompanied by loss of mitochondrial membrane potential, increased oxidative stress, structural mitochondrial disruption, and cardiomyocyte injury. These changes improved after pathway-directed intervention, suggesting that mitochondrial stress participates functionally in tissue injury amplification. From a mechanistic perspective, this is biologically coherent: mitochondrial injury may enhance reactive oxygen species production, destabilize cellular energetics, and promote inflammatory signaling, while pyroptotic membrane injury may further intensify organelle damage and inflammatory mediator release. The relationship between pyroptosis and mitochondrial dysfunction is therefore likely bidirectional and mutually reinforcing rather than strictly linear. This interpretation is consistent with the graded relationship observed between composite pyroptosis–mitochondrial scores and cardiac or systemic injury burden in the compartment-resolved experiments (17).

An important strength of the study is that the mechanistic findings were not derived from a single experimental layer. Instead, human serial biospecimens, plasma-transfer assays, tumor-bearing mouse models, and compartment-resolved intervention experiments all converged on related pathways. This cross-layer concordance strengthens the biological plausibility of the proposed framework and reduces the likelihood that the reported mechanisms are purely model-specific artifacts. At the same time, the experimental findings should still be interpreted with appropriate caution. The data support pyroptosis as a prominent and modifiable pathway, but they do not exclude the contribution of other regulated cell-death programs, such as necroptosis or ferroptosis. MODS is likely to remain a multi-mechanism process, and future work should determine how these pathways interact across organs and over time.

Several limitations should be acknowledged. First, although the clinical framework was evaluated across development and external validation cohorts, the mechanistic validation cohort included only 30 prospectively enrolled patients from a single center in China. This limits both statistical power and generalizability, particularly with respect to geographic, ethnic, and practice-pattern diversity. Second, because the study population consisted of critically ill oncology patients with complex overlapping syndromes, causal attribution of organ injury remains challenging despite structured adjudication and sensitivity analyses. Third, the study was not designed around a dedicated infection-matched comparator cohort, and therefore it cannot establish definitive diagnostic separation between immune-toxicity-associated MODS and infection-driven critical illness. Fourth, although the experimental results support pathway modulation, they do not by themselves establish immediate clinical efficacy of pyroptosis- or mitochondria-targeted treatment in human ICU practice. Future multicenter studies with diagnostically controlled designs, broader populations, and prospective intervention testing will be needed to confirm clinical applicability.

Despite these limitations, the present study has several practical implications. It suggests that critically ill patients recently exposed to checkpoint blockade may benefit from assessment frameworks that combine conventional severity scoring with immune-toxicity-related biomarker axes and phenotype-informed interpretation. Such an approach may help clinicians organize monitoring intensity, trigger earlier multidisciplinary review, and identify patients whose trajectories warrant closer evaluation for systemic immune-toxicity-associated injury. This interpretation is concordant with current guidance documents, which emphasize early recognition of severe immunotherapy-related toxicities, structured multidisciplinary assessment, and timely escalation of care in patients with suspected life-threatening organ involvement (18, 19). In particular, contemporary cardio-oncology and myocarditis recommendations support prompt cardiovascular evaluation in checkpoint inhibitor-exposed patients with hemodynamic instability, elevated cardiac biomarkers, or suspected myocarditis, given the potential for rapid deterioration and the need for coordinated diagnostic adjudication and management (20–22). In addition, emerging evidence indicates that circulating biomarkers may improve early prediction and risk stratification of immune-related adverse events, which is consistent with the biomarker-informed framework developed in the present study (23). The study therefore contributes not only a descriptive account of clinical heterogeneity but also a translational basis for future mechanism-guided critical care strategies in this population.

## 5 Conclusion

In critically ill tumor patients recently exposed to PD-(L)1 and/or CTLA-4 blockade, immune-toxicity-associated organ injury is characterized by reproducible cross-system coupling rather than isolated single-organ involvement alone. Using a framework that integrated co-occurrence analysis, phenotype discovery, outcome stratification, biomarker-informed risk modeling, and mechanistic validation, this study showed that immune-toxicity-associated MODS comprises clinically interpretable subgroups with distinct short-term mortality, organ-support burden, and ICU resource-use profiles.

The findings further suggest that adding immune-toxicity-related biomarker domains to conventional severity assessment improves early risk stratification and supports clinically structured escalation pathways. At the biological level, pyroptosis-related signaling, myeloid inflammatory amplification, endothelial–coagulation perturbation, and mitochondrial dysfunction emerged as interconnected processes associated with progression toward multi-organ injury. These results support pyroptosis and mitochondrial stress as prominent and potentially actionable components of a broader injury network, while not excluding the contribution of other regulated cell-death pathways.

Overall, this work provides a translational framework for understanding phenotypic heterogeneity and mechanistic convergence in immune-toxicity-associated critical illness. Further multicenter and diagnostically controlled studies are required to determine the generalizability of these findings and their value for guiding mechanism-informed intervention in ICU oncology practice.

## Data Availability

The raw data supporting the conclusions of this article will be made available by the authors, without undue reservation.

## References

[1] Toffart AC Meert AP Wallet F Gibelin A Guisset O Gonzalez F . ICU admission for solid cancer patients treated with immune checkpoint inhibitors. Ann Intensive Care. (2023) 13:29. doi: 10.1186/s13613-023-01122-z. PMID: 37072645 PMC10113402

[2] Lin L Houwink API van Dieren JM Wolthuis EK van Thienen JV van der Heijden MS . Treatment patterns and survival outcomes of patients admitted to the intensive care unit due to immune-related adverse events of immune checkpoint inhibitors. Cancer Med. (2024) 13:e7302. doi: 10.1002/cam4.7302. PMID: 38899457 PMC11187539

[3] Pichon S Zebian G Bureau C Levy C Lacombe C Desmedt E . Life-threatening immune-related adverse events in the intensive care unit: a narrative review. Intensive Care Med. (2025) 51:2289–304. doi: 10.1007/s00134-025-08155-x. PMID: 41123622

[4] Haanen J Obeid M Spain L Carbonnel F Wang Y Robert C . Management of toxicities from immunotherapy: ESMO Clinical Practice Guideline for diagnosis, treatment and follow-up. Ann Oncol. (2022) 33:1217–38. doi: 10.1016/j.annonc.2022.10.001. PMID: 36270461

[5] Yin Q Wu L Han L Zheng X Tong R Li L . Immune-related adverse events of immune checkpoint inhibitors: a review. Front Immunol. (2023) 14:1167975. doi: 10.3389/fimmu.2023.1167975. PMID: 37304306 PMC10247998

[6] Letcher K Johnson DB . Chronic immune-related adverse events arising from immune checkpoint inhibitors: an update. J Immunother Cancer. (2024) 12:e008591. doi: 10.1136/jitc-2023-008591. PMID: 38964785 PMC11227828

[7] van Dijk B Janssen JC van Daele PL de Jonge MJ Joosse A Verheul HM . From ICI to ICU: a systematic review of patients with solid tumors who are treated with immune checkpoint inhibitors and admitted to the intensive care unit. Cancer Treat Rev. (2025) 136:102936. doi: 10.1016/j.ctrv.2025.102936. PMID: 40222269

[8] Auch LAM Sieber C Lehnick D Hug BL . Adverse drug events of immune checkpoint inhibitors: a retrospective, descriptive real-world data analysis. BMC Cancer. (2025) 25:1303. doi: 10.1186/s12885-025-14733-5. PMID: 40790180 PMC12337401

[9] Huang C Li J Li Y Zhang C. Targeting pyroptosis for cancer immunotherapy: mechanistic insights and clinical perspectives. Mol Cancer. (2025) 24:131. doi: 10.1186/s12943-025-02344-4. PMID: 40319304 PMC12049004

[10] Sun SJ Jiao XD Chen ZG Cao Q Zhu JH Shen QR . Gasdermin-E-mediated pyroptosis drives immune checkpoint inhibitor-associated myocarditis via cGAS-STING activation. Nat Commun. (2024) 15:6640. doi: 10.1038/s41467-024-50996-5. PMID: 39103324 PMC11300882

[11] Song S Yang Y Hu Q Zhong R Lei X Wang C . Infectious adverse events associated with immune checkpoint inhibitors: a pharmacovigilance analysis based on the FAERS database. Front Immunol. (2025) 16:1647944. doi: 10.3389/fimmu.2025.1647944. PMID: 41208964 PMC12589035

[12] Itzhaki Ben Zadok O O’Hare M Nohria A . Immune checkpoint inhibitor-related myocarditis with or without concomitant myopathy: clinical findings and cardiovascular outcomes. JACC CardioOncol. (2025) 7:252–64. doi: 10.1016/j.jaccao.2025.02.005. PMID: 40246383 PMC12046767

[13] Palaskas N Siddiqui B Deswal A . Steroids in immune checkpoint inhibitor myocarditis. JACC CardioOncol. (2024) 6:800–3. doi: 10.1016/j.jaccao.2024.07.002. PMID: 39479336 PMC11520217

[14] Jensen G Wang X Kuempel J Palaskas N Chen Z Yu W . Immune checkpoint inhibitor-associated myocarditis: a historical and comprehensive review. Am J Physiol Heart Circ Physiol. (2025) 328:H734–51. doi: 10.1152/ajpheart.00687.2024. PMID: 39925096 PMC12176318

[15] Deharo F Thuny F Cadour F Resseguier N Meilhac A Gaubert M . Diagnostic value of the International Society of Cardio-Oncology definition for suspected immune checkpoint inhibitor-associated myocarditis. J Am Heart Assoc. (2023) 12:e029211. doi: 10.1016/j.acvdsp.2023.04.021. PMID: 37042287 PMC10227269

[16] Cook S Samuel V Meyers DE Stukalin I Litt I Sangha R . Immune-related adverse events and survival among patients with metastatic NSCLC treated with immune checkpoint inhibitors. JAMA Netw Open. (2024) 7:e2352302. doi: 10.1001/jamanetworkopen.2023.52302. PMID: 38236598 PMC10797458

[17] Moreno-Castaño AB Iraola G Martínez-Cibrián N Albiol N Prats DM Martinez-Sanchez J . Endothelial dysfunction and hemostatic imbalance in CAR T-cell-associated toxicities: pathophysiological insights and the role of circulating biomarkers. Front Immunol. (2025) 16:1699894. doi: 10.3389/fimmu.2025.1699894. PMID: 41256862 PMC12620366

[18] Thompson JA Schneider BJ Brahmer J Zaid MA Achufusi A Armand P . NCCN guidelines insights: management of immunotherapy-related toxicities, version 2.2024. J Natl Compr Canc Netw. (2024) 22:582–92. doi: 10.6004/jnccn.2024.0057. PMID: 39536465

[19] Aleksandrov VYZ Sagasti FMS Moreno JPS Mondéjar HH . What should intensivists know about immune checkpoint inhibitors and their side effects? Med Intensiva (Engl Ed). (2025) 49:502135. doi: 10.1016/j.medine.2025.502135. PMID: 39837744

[20] Lyon AR López-Fernández T Couch LS Asteggiano R Aznar MC Bergler-Klein J . 2022 ESC Guidelines on cardio-oncology developed in collaboration with the European Hematology Association, the European Society for Therapeutic Radiology and Oncology and the International Cardio-Oncology Society. Eur Heart J. (2022) 43:4229–361. doi: 10.1093/eurheartj/ehac244. PMID: 36017568

[21] Drazner MH Bozkurt B Cooper LT Aggarwal NR Basso C Bhave NM . 2024 ACC expert consensus decision pathway on strategies and criteria for the diagnosis and management of myocarditis: a report of the American college of cardiology solution set oversight committee. J Am Coll Cardiol. (2025) 85:391–431. doi: 10.1016/j.jacc.2024.10.080. PMID: 39665703

[22] Cheng L Xu Y Zhang S . Cardiovascular and oncological outcomes in immune checkpoint inhibitor-induced myocarditis: balancing perspectives. JACC CardioOncol. (2023) 5:745–6. doi: 10.1016/j.jaccao.2023.10.005. PMID: 38204998 PMC10774761

[23] Wang J Ma Y Lin H Cao B . Predictive biomarkers for immune-related adverse events in cancer patients treated with immune-checkpoint inhibitors. BMC Immunol. (2024) 25:8. doi: 10.1186/s12865-024-00599-y. PMID: 38267897 PMC10809515

